# A structured framework for standardized 3D leg alignment analysis: An international Delphi consensus study

**DOI:** 10.1002/ksa.12676

**Published:** 2025-04-16

**Authors:** Quinten W. T. Veerman, Gabriëlle J. M. Tuijthof, Nico Verdonschot, Reinoud W. Brouwer, Peter Verdonk, Annemieke van Haver, Hugo C. van der Veen, Peter A. J. Pijpker, Judith olde Heuvel, Roy A. G. Hoogeslag, the 3D Leg Alignment Consensus Expert Group, Ahmet Erdemir, Antoine Perrier, Bastian Sigrist, Bernardo Innocenti, Carl W. Imhauser, Claudio Belvedere, Gwendolyn Vuurberg, Harrie Weinans, Julian Fürmetz, Laura Carman, Leendert Blankevoort, Mark Taylor, Mathias Donnez, Matthias J. Feucht, Matthieu Ollivier, Michael T. Hirschmann, Min Jung, Oguzhan Tanoğlu, Philipp Niemeyer, Raghbir Khakha, Roel J. H. Custers, Ronald van Heerwaarden, Ruurd J. A. Kuiper, Sandro F. Fucentese, Steven Claes, Thor F. Besier, Vicente J. León‐Muñoz, Wolf Petersen, Wouter van Genechten, Yuanjun Teng

**Affiliations:** ^1^ OCON Centre for Orthopaedic Surgery and Sports Medicine Hengelo the Netherlands; ^2^ Department of Biomechanical Engineering, Faculty of Engineering Technology University of Twente Enschede the Netherlands; ^3^ Orthopaedic Research Laboratory, Radboud Institute for Health Sciences Radboud University Medical Center Nijmegen the Netherlands; ^4^ Department of Orthopaedic Surgery Martini Hospital Groningen Groningen the Netherlands; ^5^ Department of Orthopaedic Surgery Antwerp University Hospital Antwerp Belgium; ^6^ ORTHOCA Orthopaedic Center, AZ Monica Hospital Antwerp Belgium; ^7^ More Institute, Monica Orthopaedic Research Institute Antwerp Belgium; ^8^ Department of Orthopaedic Surgery University of Groningen, University Medical Center Groningen Groningen the Netherlands; ^9^ 3D Lab University of Groningen, University Medical Center Groningen Groningen the Netherlands; ^10^ Department of Biomedical Engineering Lerner Research Institute Cleveland Clinic OH USA; ^11^ Laboratoire TIMC‐IMAG University Grenoble Alpes, CNRS, Grenoble France; ^12^ Twinsight Grenoble France; ^13^ Center for 3D preoperative planning and 3D printing University Hospital Balgrist, University of Zurich Zurich Switzerland; ^14^ BEAMS Department Université Libre de Bruxelles Bruxelles Belgium; ^15^ Department of Biomechanics Hospital for Special Surgery New York NY USA; ^16^ Movement Analysis Laboratory, IRCCS Istituto Ortopedico Rizzoli Bologna Italy; ^17^ Department of Imaging Radboud University Medical Centre Nijmegen the Netherlands; ^18^ Department of Orthopaedics University Medical Center Utrecht Utrecht the Netherlands; ^19^ Department of Trauma Surgery BG Unfallklinik Murnau Murnau Germany; ^20^ Department of Orthopaedics and Trauma Surgery Musculoskeletal University Center Munich (MUM), University Hospital, LMU Munich Germany; ^21^ Auckland Bioengineering Institute The University of Auckland New Zealand; ^22^ Department of Orthopaedic Surgery and Sports Medicine Amsterdam UMC location University of Amsterdam Amsterdam the Netherlands; ^23^ Amsterdam Movement Sciences, Musculoskeletal Health Amsterdam the Netherlands; ^24^ Medical Device Research Institute, College of Science and Engineering Flinders University Adelaide Australia; ^25^ Newclip Technics, Haute‐Goulaine France; ^26^ Department of Orthopedic Surgery and Traumatology Freiburg University Hospital, Albert Ludwigs University Freiburg Freiburg Germany; ^27^ Aix Marseille Univ, CNRS, ISM Marseille France; ^28^ Department of Orthopaedic Surgery and Traumatology Kantonsspital Baselland Bruderholz Switzerland; ^29^ Department of Clinical Research, Research Group Michael T. Hirschmann, Regenerative Medicine & Biomechanics University of Basel Basel Switzerland; ^30^ Department of Orthopaedic Surgery, Arthroscopy and Joint Research Institute Yonsei University College of Medicine Seoul Republic of Korea; ^31^ Department of Orthopaedics and Traumatology İzmir Democracy University İzmir Turkey; ^32^ OCM, Orthopädische Chirurgie München Munich Germany; ^33^ Department of Trauma and Orthopaedics Guys and St Thomas Hospital London United Kingdom; ^34^ Centre for Deformity Correction and Joint Preserving Surgery Kliniek ViaSana Mill The Netherlands; ^35^ Image Sciences Institue University Medical Center Utrecht Utrecht the Netherlands; ^36^ Department of Orthopaedics and Traumatology University Hospital Balgrist, University of Zurich Zurich Switzerland; ^37^ Orthopedic Department AZ Herentals Hospital Antwerp Belgium; ^38^ Department of Engineering Science & Biomedical Engineering University of Auckland New Zealand; ^39^ Instituto de Cirugía Avanzada de la Rodilla (ICAR) Murcia Spain; ^40^ Department of Surgery Paediatrics and Obstetrics & Gynaecology (Faculty of Medicine; Murcia University) Murcia Spain; ^41^ Department of Orthopaedic Surgery and Traumatology Hospital General Universitario Reina Sofía Murcia Spain; ^42^ Klinik für Orthopädie und Unfallchirurgie, Martin Luther Hospital Berlin Germany; ^43^ Orthopaedic department University hospital Antwerp Antwerp Belgium; ^44^ Academy for Engineering and Technology Fudan University Shanghai China; ^45^ Department of Orthopaedic Surgery, Huashan Hospital Fudan University Shanghai China

**Keywords:** alignment parameters, consensus, coordinate systems, joint orientation, knee

## Abstract

**Purpose:**

To reach consensus among international experts on a structured framework for standardized 3D leg alignment analysis based on 3D bone models, ensuring consistency and improving clinical applicability.

**Methods:**

A Delphi study was performed in four rounds. Rounds 1 and 2 involved a steering and rating group that developed statements based on principles preserving the 3D complexity of anatomical structures, identified through a systematic review. These statements encompassed approaches for deriving joint centres and joint orientations, and defining coordinate systems using 3D bone models. In Rounds 3 and 4, a panel of 35 international experts, including clinicians (54%) and engineers (46%), with participants from Europe (80%), Oceania (9%), Asia (6%), and the Americas (6%), evaluated these statements. Consensus was defined as ≥80% agreement.

**Results:**

Rounds 1 and 2 resulted in 31 statements to be included in the survey. Of these, 26 achieved consensus in Round 3, with the five remaining statements refined and reaching consensus in Round 4. Experts agreed on utilising all available relevant surface data to define joint centres, joint orientations, and individual femoral and tibial coordinate systems alongside a combined leg coordinate system, and adopting central 3D axes for femoral version and tibial torsion.

**Conclusions:**

This international Delphi consensus study provides a structured framework for a standardized 3D leg alignment analysis based on 3D bone models. This framework aims to enhance clinical applicability for preoperative planning and execution of uni‐ and multiplanar correction osteotomies around the knee, reduce the methodological variability in 3D leg alignment analysis literature, and improve cross‐study comparability.

**Level of Evidence:**

Level V.

**Abbreviations**
2D: Two‐dimensional
3D: Three‐dimensional
4D: Four‐dimensional
CT: Computed Tomography
ISB: International Society of Biomechanics

## INTRODUCTION

Three‐dimensional (3D) bone models are increasingly adopted for leg alignment analysis [1, 9, 20, 22]. Unlike the traditional two‐dimensional (2D) framework that is subject to errors due to image distortion and inadequate leg and knee positioning [18, 23, 25], the use of 3D bone models enables a more accurate assessment of multiplanar deformities without oversimplifying the complexity of reality [16, 30]. This advancement has laid the foundation of several proposed clinical workflows [12, 17, 19, 29]

Although the approach to defining leg alignment parameters and the coordinate systems in which alignment parameters are expressed is critical, a recent systematic review [31] identified substantial variability in the methods and underlying principles used to derive axes and joint orientations from 3D bone models. Such variability may result in considerable differences in alignment parameter values across studies, even when the same anatomical reference planes and coordinate systems are used. Furthermore, these discrepancies are likely to be amplified if the definitions of coordinate systems themselves differ between studies. Consequently, comparing outcomes across studies becomes challenging, and the ‘normal values’ for alignment parameters reported using 3D models may only be valid within the specific contexts of those studies, limiting their broader applicability. This highlights the need for a unified approach to enhance reliability and consistency.

Despite these inconsistencies, several underlying principles for deriving axes and joint orientations from 3D bone models have been identified that preserve the complexity of anatomical structures in 3D bone models. These include using centroids of marked articular surface data to determine joint centres, geometrical shape‐fitting to determine joint centres and joint orientations of articular surfaces that are more round, and plane‐fitting to determine joint orientations of articular surfaces that are more flat [31]. Establishing consensus on these principles might be a crucial step towards developing a unified 3D framework for leg alignment analysis.

Therefore, the purpose of this study was to determine whether consensus could be reached on a structured framework for standardized 3D leg alignment analysis based on 3D bone models, ensuring consistency and improving clinical applicability. The aim of this study was to quantify the level of agreement on principles for obtaining leg axes, joint orientations and coordinate systems, with the potential to standardize clinical workflows and enhance surgical planning.

## MATERIALS AND METHODS

**Figure 1:**
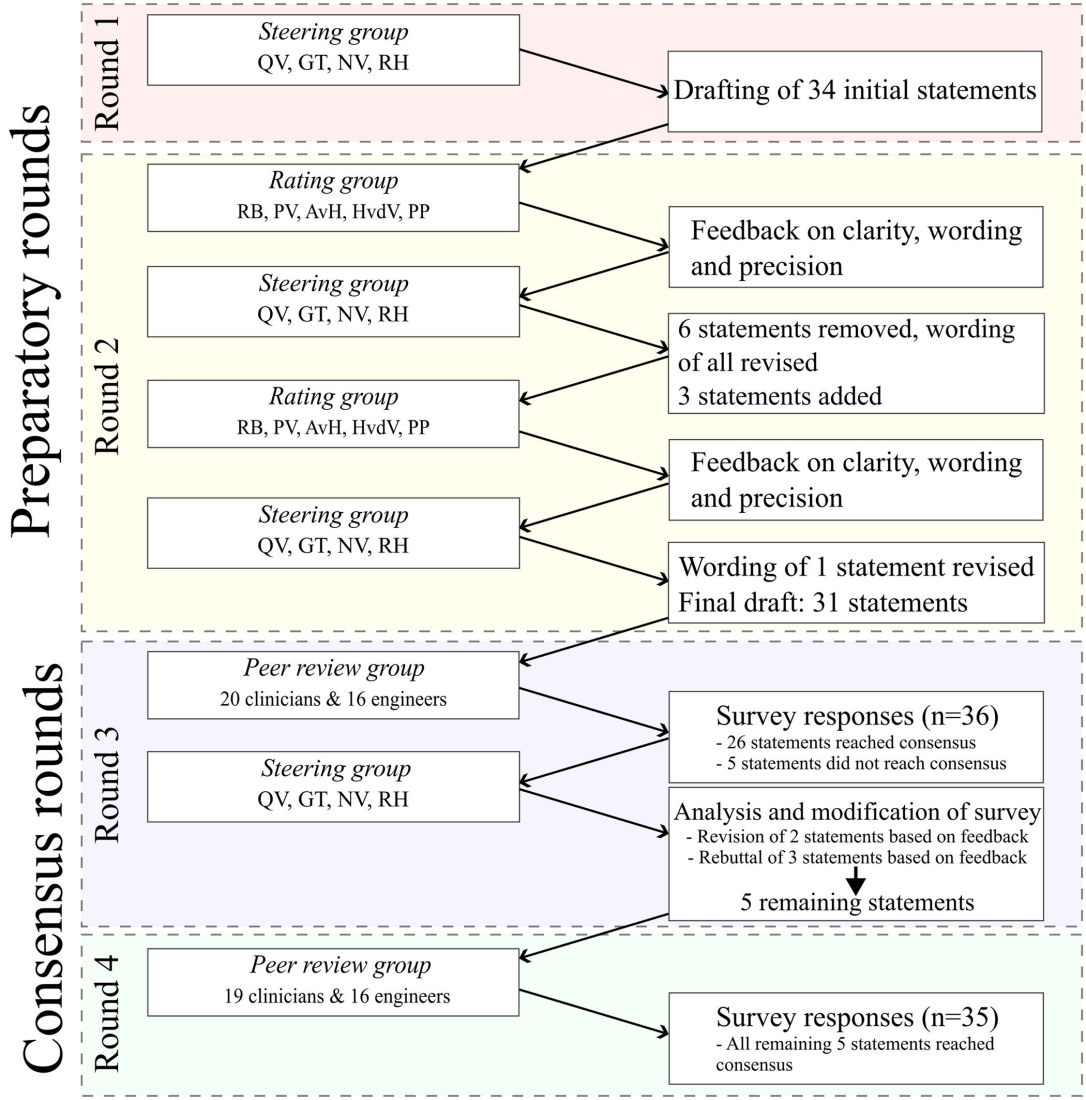
Flowchart visualizing all four rounds of this Delphi consensus study. Steering and rating group described using authors’ initials. AvH, Annemieke van Haver; GT, Gabriëlle Tuijthof; HvdV, Hugo van der Veen; NV, Nico Verdonschot; PP, Peter Pijpker; PV, Peter Verdonk; QV, Quinten Veerman; RB, Reinoud Brouwer; RH, Roy Hoogeslag.

An e‐Delphi method [3, 7, 11, 21, 24, 32] with four rounds was conducted to systematically achieve consensus among international experts while maintaining anonymity, thereby preventing conformity bias. Consensus was defined a priori as ≥80% agreement using a binary agree/disagree scale with an open‐text comment field. This combined quantitative and qualitative approach was chosen to ensure clear acceptance or rejection of statements while allowing participants to provide detailed feedback for iterative refinement of both content and wording. The first two preparatory rounds (Rounds 1 and 2) focused on developing and refining statements, while the subsequent two rounds (Rounds 3 and 4) aimed to validate these statements through expert consensus (Figure 1).

### Rounds 1 and 2: Statement development

In Round 1, a steering group was formed based on existing collaborations between an orthopaedic surgeon and technical physician of *OCON Centre for Orthopaedic Surgery and Sports Medicine* and two engineers of the Department of Biomechanical Engineering at *University of Twente*. This group, comprising four co‐authors: *QV, GT, NV, RH*, developed an initial set of statements (Round 1) based on a comprehensive systematic review of the literature of 93 studies (January 2006 – June 2024) on methods for deriving axes and joint orientations from 3D bone models for knee‐related leg alignment analysis. The review identified fundamental principles that preserve the complexity of anatomical structures, which were incorporated into the statements. The results of the systematic review were recently published and were available for all panel members of the present study [31]. This set of statements covered five key categories: 1) joint centres; 2) individual femoral and tibial/fibular coordinate systems; 3) combined femoral and tibial/fibular (leg) coordinate system; 4) joint orientations; 5) femoral version and tibial torsion.

Each statement was illustrated with an example such as centroid calculation of marked articular surface data to define joint centres, geometrical shape‐fitting to define joint centres and joint orientations of articular surfaces that are more round, and plane‐fitting to define joint orientations of articular surfaces that are more flattter [31] (see Supporting Information A). These examples clarified principles without prescribing a specific method or debate specific technical implementations, allowing freedom to utilize tools available to the analyst and leaving room for future technical developments.

In Round 2, the statements were electronically distributed to the rating group of five international European expert orthopaedic surgeons and engineers, recruited from the steering groups’ professional network (co‐authors: *RB, PV, AvH, HvdV, PP*). Participants evaluated each statement on whether it should be included and were encouraged to provide free‐text comments to capture additional feedback and refinements. The statements were iteratively refined with controlled anonymous feedback to the rating group. This process continued until all rating group members agreed on clarity, wording, and precision of the statements. Although selected from the steering group's network, the rating group included experts from different institutions and disciplines, ensuring an independent evaluation process.

### Rounds 3 and 4: Delphi consensus process

#### Expert panel selection

For Rounds 3 and 4, international experts were invited to join a peer review group (3D leg alignment consensus expert group). Inclusion criteria were: 1) Academic contributions: authors who had made significant contributions to the literature on 3D leg alignment analysis, as identified in a recently published systematic review; [31] 2) Field recognition: surgeons and engineers recognized in the field of 3D‐planned osteotomies around the knee (amongst those all steering group members of the *ESSKA* consensus guidelines on the painful degenerative varus knee [8, 26]); 3) Referrals: referrals by the already‐invited experts; and 4) Prior involvement: members of the rating group. Steering group members were excluded from the surveys to mitigate moderator bias.

To enhance geographical adaptability and generalizability of the consensus, experts from various continents were invited. To avoid redundancy, only the first and senior authors from distinct research groups were included.

If initial email invitations were unanswered, up to three reminders were sent over four weeks to maximize participation.

Of 68 eligible experts invited to participate in the peer review group (Rounds 3 and 4), 36 (53%) agreed to take part in the Delphi study, including five from the rating group. The panel comprised 19 (54%) clinicians and 16 (46%) engineers. Response rates varied across regions (Asia, 18%; Europe, 58%; Americas, 67%; Oceania, 75%). Most participants were from Europe (28/35; 80%), followed by Oceania (3/35; 9%), Asia (2/35; 6%), and the United States of America (2/35; 6%). The participants responded to the two rounds of surveys between June and July 2024. Response rates remained high throughout, with 97% (35/36) completing Round 4.

#### Consensus evaluation

The finalized statements were distributed to the expert panel via an online survey platform (SurveyMonkey Inc., San Mateo, California, USA) [24]. In Round 3, participants evaluated each statement using the 80% agreement threshold [15, 32], and free‐text comments were encouraged. Statements that did not meet this 80% consensus threshold were presented again in Round 4, along with all unedited comments from Round 3. Each remaining statement was either modified (with a reply) or left unchanged (with a rebuttal) before re‐evaluation in Round 4. To maintain full transparency, all experts had access to the original Round 3 comments along with structured justifications for any modifications or rebuttals, ensuring that refinements were based on collective expert input rather than isolated feedback (Supplementary Information A2). Experts were acknowledged as co‐authors only if they completed both rounds of the consensus process.

### Data collection and statistical analysis

Demographic data, including country of practice, years of experience, and field of expertise were collected from all participants and presented as absolute numbers and percentages. The degree of consensus was expressed as the percentage of participants agreeing with the statement. Experts who completed Round 3 but not Round 4 were included only in the Round 3 consensus results.

## RESULTS

### Rounds 1 and 2

The steering group developed 34 statements in Round 1, assessed twice by the rating group in Round 2. After the first assessment, six statements were removed. The wording of all statements was revised, and three new statements were added. After a second assessment, one statement was further modified (Figure 1). Subsequently, the rating group agreed on the clarity, wording, and precision of the final 31 statements.

### Rounds 3 and 4

Of the 31 statements presented in Round 3, 26 (84%) reached consensus, while the remaining five were refined based on iterative feedback and reached consensus in Round 4 (Figure 1; Table 1).

**Table 1:** Level of agreement per presented statement. Statements not reaching at least 80% in Round 3 were modified or had their context updated and were presented again in Round 4. An overview of each statement with the presented illustrative example and given and/or updated context is presented in Supporting Information A.

Statement	%‐agreement Round 3 (*n* = 36)	%‐agreement Round 4 (*n* = 35)
**Joint centers**		
The proximal femoral joint centre is derived from all available surface data of the articular surface of the femoral head.	91.7%	‐
The distal femoral joint centre is derived from all available surface data of the articular surface of the medial and lateral distal femoral condyles.	72.2%^a^	91.4%
The articular surface of the distal femur is divided into three segments:	83.3%	‐
a. the medial femoral condyle;		
b. the lateral femoral condyle;		
c. and the trochlea.		
The femoral condyles and trochlea are divided at the level of the sulcus terminalis, and the medial and lateral femoral condyles are divided at the deepest point of the trochlea and/or the highest anterior point of the notch.		
The proximal tibial joint centre is derived from all available surface data of the articular surface of the medial and lateral tibial plateau.	86.1%	‐
The distal tibial/fibular joint centre is derived from all available surface data of the combined distal tibial and fibular articular surfaces.	83.3%	‐
**Individual femoral and tibial/fibular coordinate systems**		
Conform the ISB recommendations, the distal‐proximal axis is the y‐axis, and points cranially; the medial‐lateral axis is the z‐axis, and points laterally; and the posterior‐anterior axis is the x‐axis, and points anteriorly.	77.8%^b^	‐
*Modified for Round 4 to:*	‐	85.7%
The medial‐lateral axis is the x‐axis, and points to the right; the posterior‐anterior axis is the y‐axis, and points anteriorly; and the distal‐proximal axis is the z‐axis, and points cranially.		
The femoral coordinate system's origin is coincident with the distal femoral joint centre.	94.4%	‐
The femoral coordinate system's distal‐proximal axis is parallel with the mechanical femoral axis.	91.7%	‐
The femoral coordinate system's medial‐lateral axis is parallel to the projection (in the direction of the mechanical femoral axis) of the medial‐lateral axis of the distal femur on the femoral axial plane.	80.6%	‐
The femoral coordinate system's posterior‐anterior axis is orthogonal to the other two axes.	97.2%	‐
For the femoral coordinate system, the medial‐lateral axis of the distal femur is derived from all available surface data of the articular surface of the medial and lateral distal femoral condyle.	86.1%	‐
The tibial/fibular coordinate system's origin is coincident with the proximal tibial joint center.	91.7%	‐
The tibial/fibular coordinate system's distal‐proximal axis is parallel to the mechanical tibial axis.	88.9%	‐
The tibial/fibular coordinate system's medial‐lateral axis is parallel to the projection (in the direction of the mechanical tibial axis) of the medial‐lateral axis of the proximal tibia on the tibial axial plane.	88.9%	‐
The tibial/fibular coordinate system's posterior‐anterior axis is orthogonal to the other two axes.	97.2%	‐
For the tibial coordinate system, the medial‐lateral axis is derived from all available surface data of the articular surface of the medial and lateral tibial plateau.	91.7%	‐
**Combined femoral and tibial/fibular (leg) coordinate system**		
To define the leg coordinate system, it is a prerequisite that the knee is extended, meaning that the mechanical femoral and tibial axes are parallel to each other (in the sagittal plane of the leg coordinate system).	77.8%^b^	‐
*Modified for Round 4 to:*	‐	88.6%
To define distal femoral and proximal tibial joint orientation angles (i.e., the projection of the distal femoral and proximal tibial joint orientation and the relevant longitudinal axes on the coronal plane of the leg coordinate system), it is a prerequisite that the knee is extended; meaning that the mechanical femoral and tibial axes are parallel to each other (in the sagittal plane of the leg coordinate system).		
The origin is coincident with the distal femoral joint centre.	86.1%	‐
The distal‐proximal axis is parallel to the mechanical leg axis.	91.2%	‐
The medial‐lateral axis is parallel to the projection of the medial‐lateral axis of the distal femur on the leg axial plane, in the direction of the mechanical leg axis.	83.3%	‐
The posterior‐anterior axis is orthogonal to the other two axes.	97.2%	‐
**Joint orientations**		
The distal femoral condylar joint orientation is based on the most distal point(s) (relative to a reference coordinate system; part 3 of the survey) of the articular surfaces of the medial and lateral femoral condyle.	86.1%	‐
The femoral supracondylar‐trochlear orientation is based on the most proximal borders (relative to a reference coordinate system; part 3 of the survey) of all available surface data of the articular surfaces of the trochlea and medial and lateral femoral condyles.	83.3%	‐
The medial tibial plateau's joint orientation is derived from all available surface data of the medial tibial plateau's articular surface.	94.4%	‐
The lateral tibial plateau's joint orientation is derived from all available surface data of the lateral tibial plateau's articular surface.	97.2%	‐
The tibial plateau's joint orientation is derived from all available surface data of the medial and lateral tibial plateau's articular surface.	88.9%	‐
**Femoral version and tibial torsion**		
For femoral version, the medial‐lateral joint orientation of the proximal femur is based on a line connecting the proximal femoral joint centre to the centre of the femoral neck.	97.2%	‐
For femoral version, the medial‐lateral joint orientation of the distal femur is based on the central distal femoral axis which is derived from all available surface data of the articular surface of the medial and lateral distal femoral condyles.	75.0%^a^	91.4%
For tibial torsion, the medial‐lateral joint orientation of the proximal tibia is based on the central proximal tibial axis which is derived from all available surface data of the articular surface of the medial and lateral tibial plateau.	77.8%^a^	88.6%
For tibial torsion, the medial‐lateral joint orientation of the distal tibia/fibula is based on the intermalleolar axis which is derived from all available surface data the articular surfaces of the medial and lateral malleolus.	80.6%	‐
The articular surface of the distal tibial/fibular is divided into three segments:	91.7%	‐
a. the medial malleolus;		
b. the lateral malleolus;		
c. and the tibial plafond.		
The tibial plafond and the medial malleolus are divided at the level of the medial gutter.		

^a^ The context of these statements were updated between Rounds 3 and 4. The steering group rebutted each provided comment of the participants and provided additional argumentation, clarification or examples before asking the initial statement again.

^b^ These statements were modified between Rounds 3 and 4 based on the provided comments of the participants.

### Joint centres

**Figure 2:**
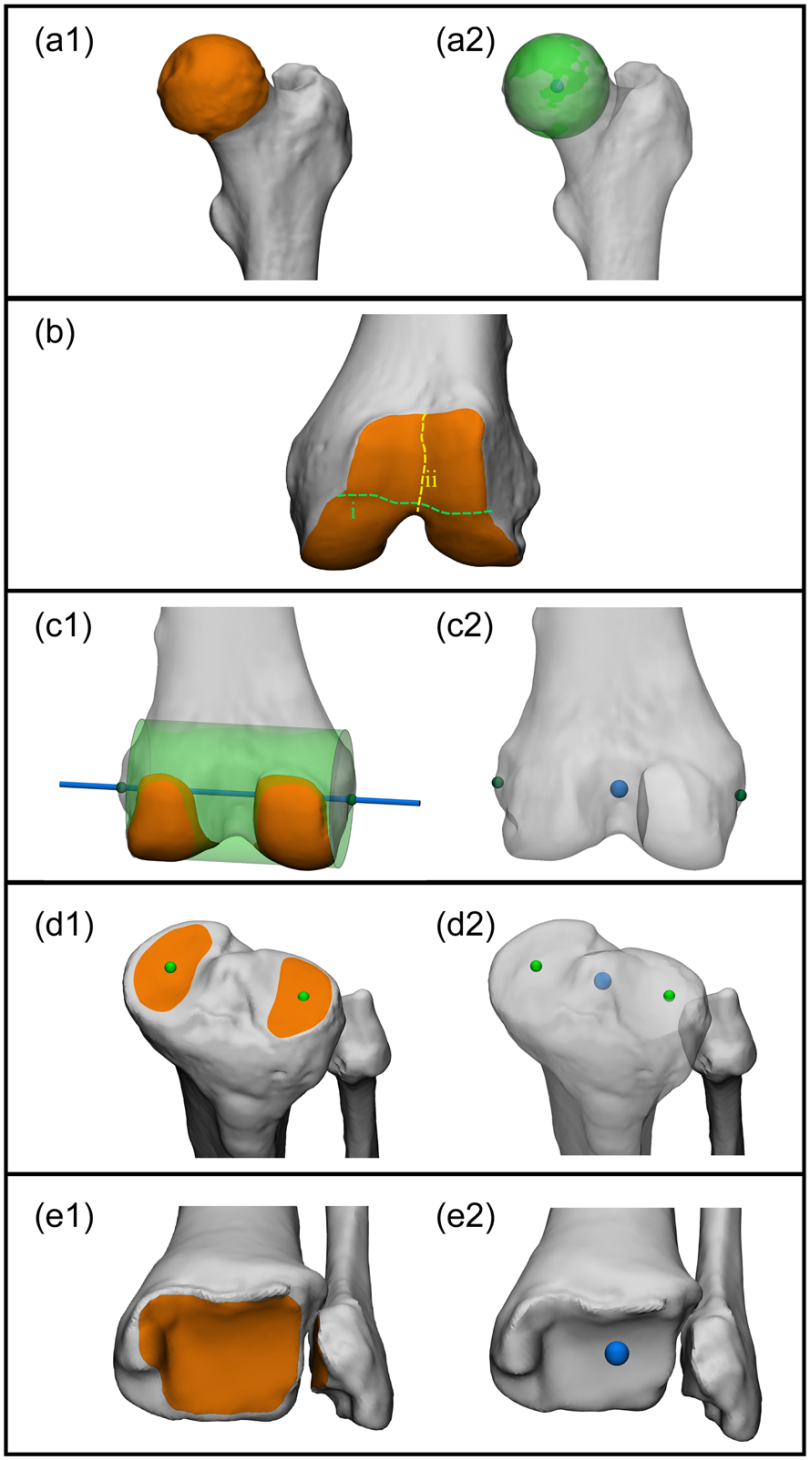
Illustrative example of clinical application of leg alignment analysis on 3D bone models based on the agreed upon principles in the present study: defining joint centers. (a) Anterior view of a left proximal femur. (a1) Identification of the articular surface of the femoral head (orange). (a2) Best fit geometrical shape (green sphere) to the articular surface. The proximal femoral joint centre: the calculated centroid of the best fit sphere (blue). The centroid and the joint centre overlap. (b): Anterior view of a left distal femur. Since the tibiofemoral and patellofemoral joint surfaces are continuous, for 3D leg alignment analysis the sulcus terminalis (i; green dashed) separates the condyles from the trochlea, and the trochlear groove line (ii; yellow dashed) separates the medial from the lateral condyle. (c): Posterior view of a left distal femur. (c1) Identification of the articular surfaces of the medial and lateral condyles (orange). Best fit geometrical shape (green cylinder) to the distal femoral condyles articular surface, and calculation of the cylinder's central axis (blue line) and its intersections with the femur (dark green spheres). (c2) The distal femoral joint centre: midpoint (blue sphere) of the intersections with the femur (green spheres). (d): Anterolateral view of a left proximal tibia and fibula. (d1) Identification of the articular surfaces of the medial and lateral tibial plateaus (orange), and centroid calculation (green spheres) of each articular surface. The proximal tibial/fibular joint centre: midpoint (blue sphere) of the calculated centroids (green spheres). (e): Anterior view of a left distal tibia and fibula. (e1) Identification of the articular surfaces of the tibial plafond and medial and lateral malleolus (orange). (e2) The distal tibial/fibular joint centre: the centroid (blue sphere) to the identified articular surfaces. The centroid and the joint centre overlap. With the definition of joint centre, the mechanical axes of the femur, tibia, and leg can be established.

Accurate determination of hip, knee and ankle joint centres to define mechanical axes is considered a critical aspect in deformity correction. The expert panel reached consensus that joint centres in 3D should be derived from all available articular surface data of the 3D bone models (Figure 2). Given the continuous nature of the tibiofemoral and patellofemoral joint surfaces, the expert panel agreed to divide the distal femoral joint surface at the level of the trochlea and the medial and lateral femoral condyles (Figure 2b).

### Individual femoral and tibial/fibular coordinate systems

**Figure 3:**
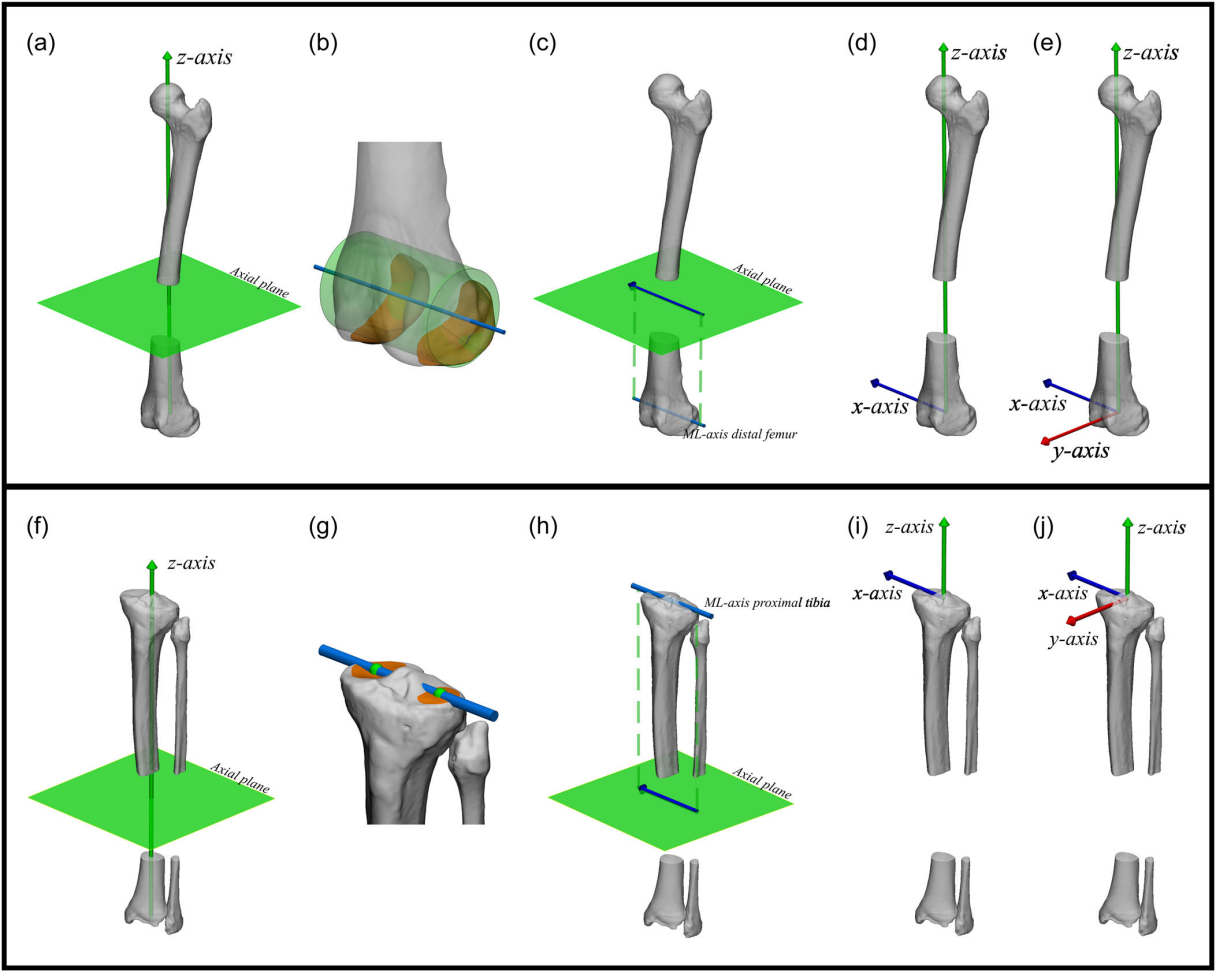
Illustrative example of clinical application of leg alignment analysis on 3D bone models based on the agreed upon principles in the present study: defining femoral and tibial/fibular coordinate systems. Top: Anterolateral view of a left femur. Femoral coordinate system (a‐e). (a) The direction of the distal‐proximal axis (z‐axis; green arrow) is defined to be coincident with the mechanical femoral axis, thereby also defining the axial plane (green plane). (b) The central medial‐lateral axis of the distal femur: the central axis (blue line) of a best fit geometrical shape (green cylinder) to the articular surface of the distal femoral condyles (orange). (c) The direction of the medial‐lateral axis (x‐axis; blue arrow) is defined coincident with the central medial‐lateral axis of the distal femur (cyan line), projected on the axial plane along the direction of the mechanical femoral axis (green dashed). The x‐axis points to the right. (d) The resulting x‐ and z‐axis defined from the origin (distal femoral joint center). (e) The posterior‐anterior axis (y‐axis; red arrow) is orthogonal to both the x‐ and z‐axis. Bottom: Anterolateral view of the tibial/fibula. Tibial/fibular coordinate system (f‐j). (f) The direction of the distal‐proximal axis (z‐axis; green arrow) is defined to be coincident with the mechanical tibial axis, thereby also defining the axial plane (green plane). (g) The central medial‐lateral axis of the proximal tibia: line (blue line) connecting the centroids (green spheres) to the articular surfaces of the medial and lateral tibial plateau (orange). (h) The direction of the medial‐lateral axis (x‐axis; blue arrow) is defined coincident with the central medial‐lateral axis of the proximal tibia (cyan line), projected on the axial plane along the direction of the mechanical tibial axis (green dashed). The x‐axis points to the right. (i) The resulting x‐ and z‐axis defined from the origin (proximal tibial joint center). (j) The posterior‐anterior axis (y‐axis; red arrow) is orthogonal to both the x‐ and z‐axis. ML, medial‐lateral.

For purpose of reference and clinical utility, axes and joint orientations are projected onto coronal, sagittal or axial anatomical reference planes. Therefore, defining a coordinate system becomes essential. The expert panel reached consensus on definitions for both femoral and tibial/fibular coordinate systems (Figure 3). The expert panel further agreed that the medial‐lateral axes for both coordinate systems should be derived from all available articular surface data of the medial and lateral distal femoral condyles and tibial plateaus, respectively (Figure 3b,g).

### Combined femoral and tibial/fibular (leg) Coordinate System

**Figure 4:**
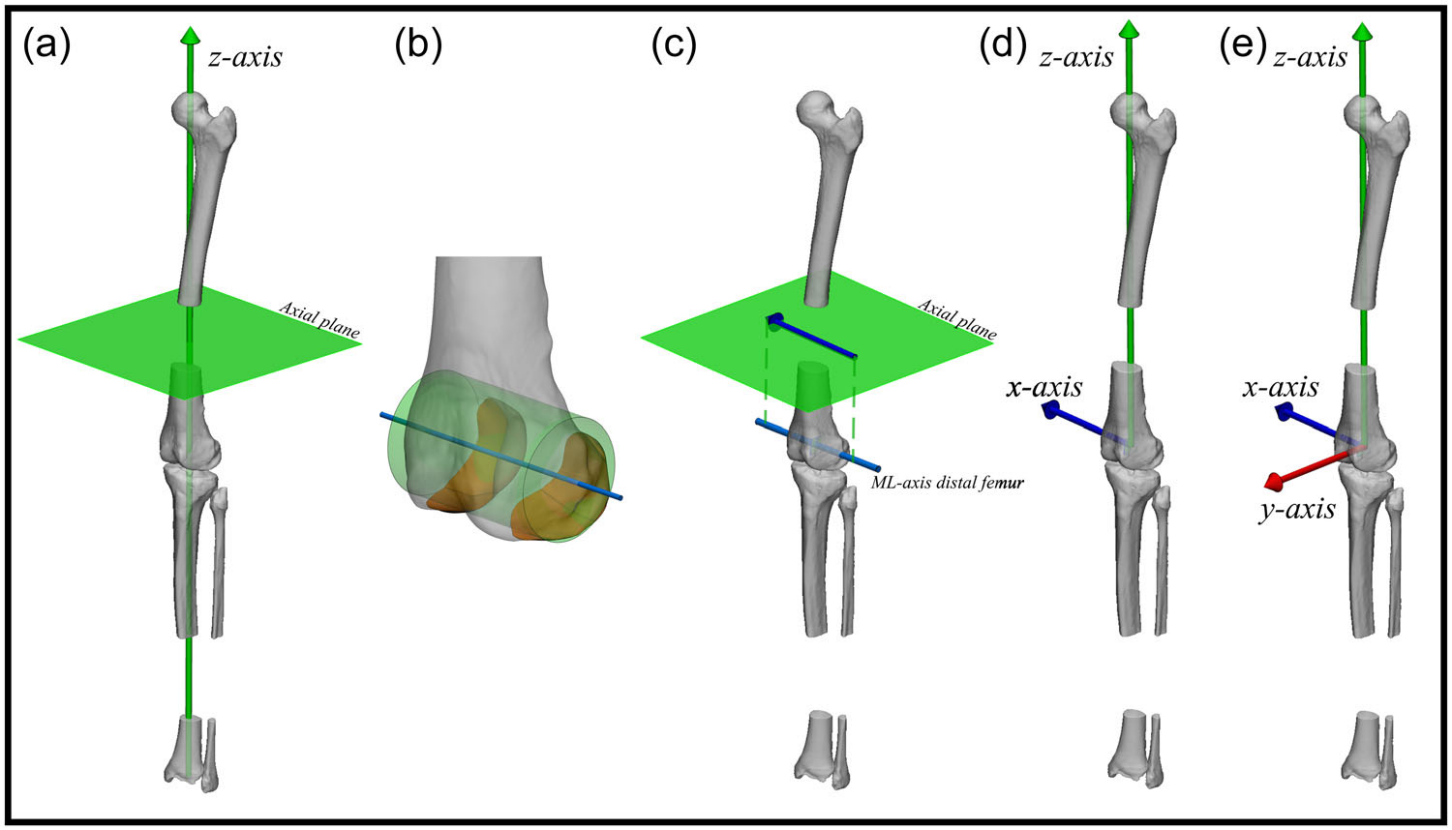
Illustrative example of clinical application of leg alignment analysis on 3D bone models based on the agreed upon principles in the present study: defining a combined femoral and tibial/fibular (leg) coordinate system. Anterolateral view of a left leg. Combined femoral and tibial/fibular (leg) coordinate system (a‐e). (a) The direction of the distal‐proximal axis (z‐axis; green arrow) is defined to be coincident with the mechanical leg axis, thereby also defining the axial plane (green plane). (b) The central medial‐lateral axis of the distal femur: the central axis (blue line) of a best fit geometrical shape (green cylinder) to the articular surface of the distal femoral condyles (orange). (c) The direction of the medial‐lateral axis (x‐axis; blue arrow) is defined coincident with the central medial‐lateral axis of the distal femur (cyan cylinder), projected on the axial plane along the direction of the mechanical leg axis (green dashed). The x‐axis points to the right. (d) The resulting x‐ and z‐axis defined from the origin (distal femoral joint center). (e) The posterior‐anterior axis (y‐axis; red arrow) follows from being orthogonal to both the x‐ and z‐axis. ML, medial‐lateral.

Given that joint alignment (i.e., the collinearity of the joint centres of the hip, knee and ankle) is expressed in the coronal plane of the leg, the expert panel reached consensus on a definition for a leg coordinate system in addition to the femoral and tibial coordinate systems (Figure 4).

### Joint orientations

**Figure 5:**
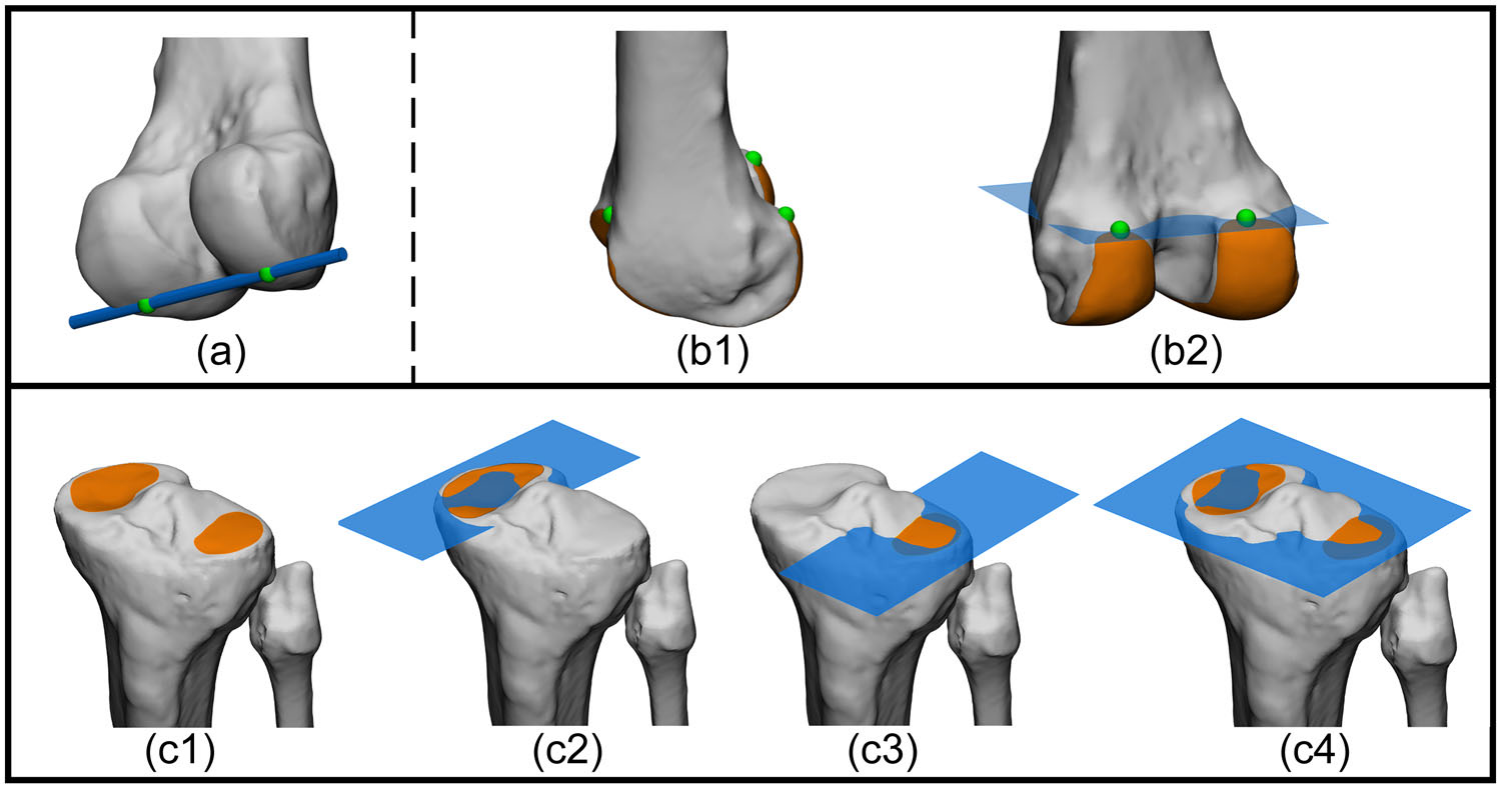
Illustrative example of clinical application of leg alignment analysis on 3D bone models based on the agreed upon principles in the present study: defining joint orientations. (a) Posteromedial view of a left distal femur. The distal femoral condylar joint orientation: a tangent line (blue) connecting the most distal points (green; relative to a reference coordinate system*) of all available surface data of the medial and lateral femoral condyle articular surfaces. (b) Lateral (b1) and posterior (b2) view of a left distal femur. (b1) Identification of the most proximal points (green spheres; relative to a reference coordinate system*) of all available articular surface data of the trochlea and medial and lateral femoral condyles (orange). (b2) The supracondylar femoral joint orientation: a plane (blue) connecting the identified most proximal points. (c) Anterolateral view of a left proximal tibia/fibula. (c1) Identification of the articular surfaces of the medial and lateral tibial plateaus (orange). (c2) Medial tibial plateau joint orientation: a plane (blue) fit through the articular surface of the medial tibial plateau (orange). (c3) Lateral tibial plateau joint orientation: a plane (blue) fit through the articular surface of the lateral tibial plateau (orange). (c4) Tibial plateau joint orientation: a plane (blue) fit through the articular surfaces of both the medial and lateral tibial plateau (orange). *, can only be established after the relevant coordinate system is defined.

The expert panel agreed that joint orientations in 3D should also be derived from all available articular surface data of the 3D bone models (Figure 5). The distal femoral condylar (Figure 5a) and supracondylar (Figure 5b) joint orientations depended on the most distal and proximal points relative to a prerequisite coordinate system, respectively, of all available articular surface data.

### Femoral version and tibial torsion

**Figure 6:**
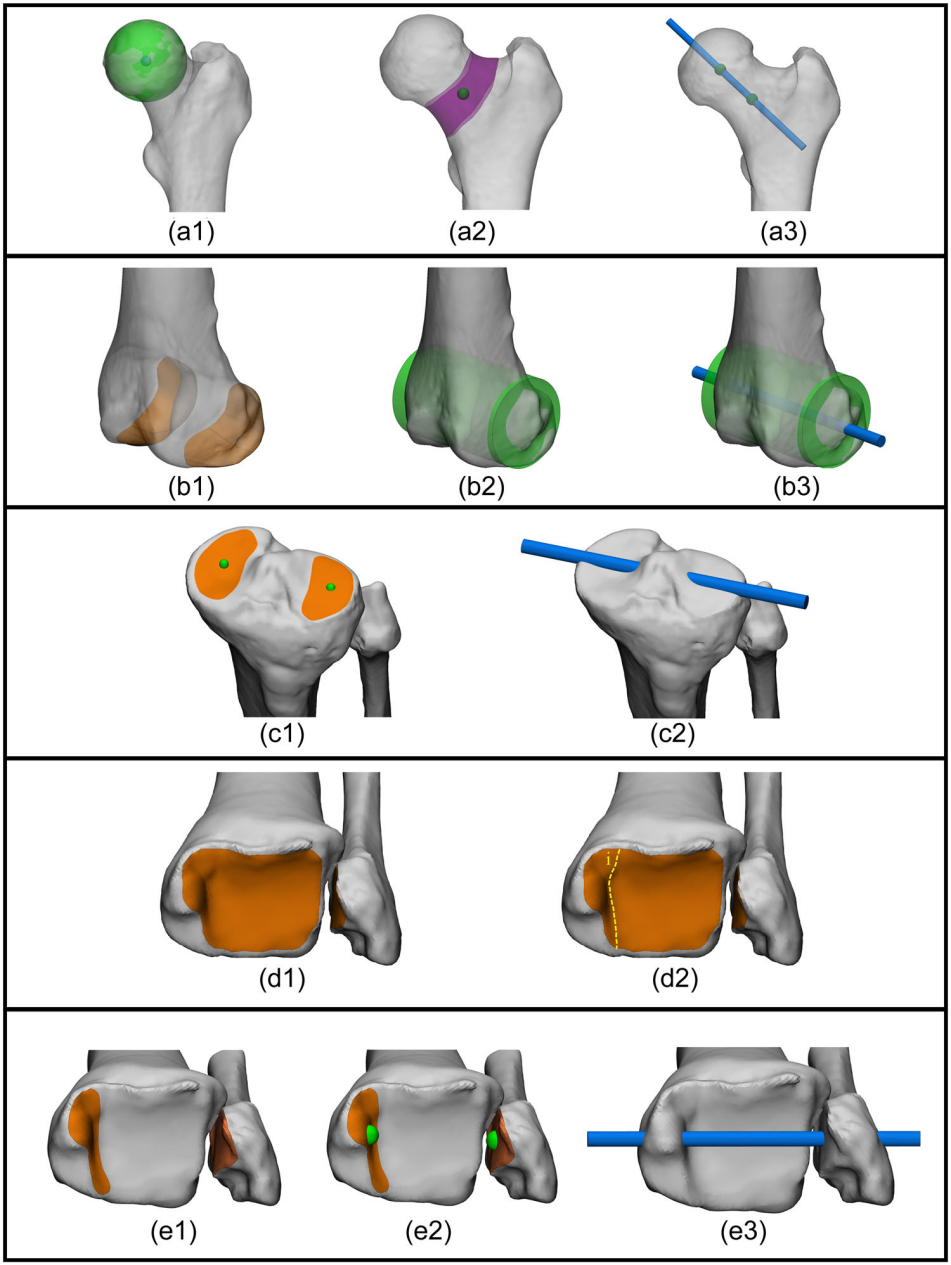
Illustrative example of clinical application of leg alignment analysis on 3D bone models based on the agreed upon principles in the present study: defining the proximal and distal medial‐lateral axis for measurement of femoral version and tibial torsion. (a) Anterior view of a left proximal femur. (a1) The proximal femoral joint centre: the centroid (blue) of the best fit geometrical shape (green sphere) to the articular surface of the femoral head. The centroid and the joint centre overlap. (a2) The femoral neck centre: the centroid (green) of all available surface data of the femoral neck (purple). (a3) The central medial‐lateral axis of the proximal femur (the neck‐femur axis): line (blue line) connecting the proximal femoral joint centre and the femoral neck centre (green spheres). (b) Anterolateral view of a distal femur. (b1) Identification of the articular surfaces of the medial and lateral condyles (orange). (b2) Best fit geometrical shape (green cylinder) to the distal femoral condyles articular surface, and calculation of the cylinder's central axis (blue line). (b3) The central medio‐lateral axis of the distal femur is defined to be coincident with this central axis. (c): Anterolateral view of a left proximal tibia and fibula. (c1) Identification of the articular surfaces of the medial and lateral tibial plateaus (orange), and centroid calculation (green spheres) of each articular surface. (c2) The central medio‐lateral axis of the proximal tibia: line (blue line) connecting the centroids of each articular surface. (d): Anterior view of a left distal tibia/fibula. Since the tibial plafond and medial malleolus joint surfaces (orange, d1) are continuous by nature, for 3D leg alignment analysis, these are separated at the level of the medial gutter (i; yellow dashed, d2). (e): Anterior view of a left distal tibia/fibula. (e1) Identification of the articular surfaces of the medial and lateral malleolus (orange). (e2) Calculated centroids of the identified articular surfaces (green spheres). (e3) The central medial‐lateral axis of the distal tibia/fibula (the intermalleolar axis): line connecting the centroids of the medial and lateral malleolus (blue line).

Consensus was reached that 3D femoral version and tibial torsion should be determined from the central medial‐lateral axes of both the proximal and distal joints (Figure 6). Additionally, the panel agreed that these central medial‐lateral axes should be derived from all available relevant surface data. For the distal femur and proximal tibia, this corresponds to the medial‐lateral axes that define the coronal plane within their respective coordinate systems (Figure 6b,c). For the distal tibia/fibula, the panel agreed that the medial‐lateral axis should be derived using all available articular surface data of the medial and lateral malleoli (Figure 6e). Given the continuous nature of the tibial plafond and medial malleolus articular surfaces, the expert panel agreed to divide these at the level of the medial gutter (Figure 6d).

### 3D leg alignment analysis

**Figure 7:**
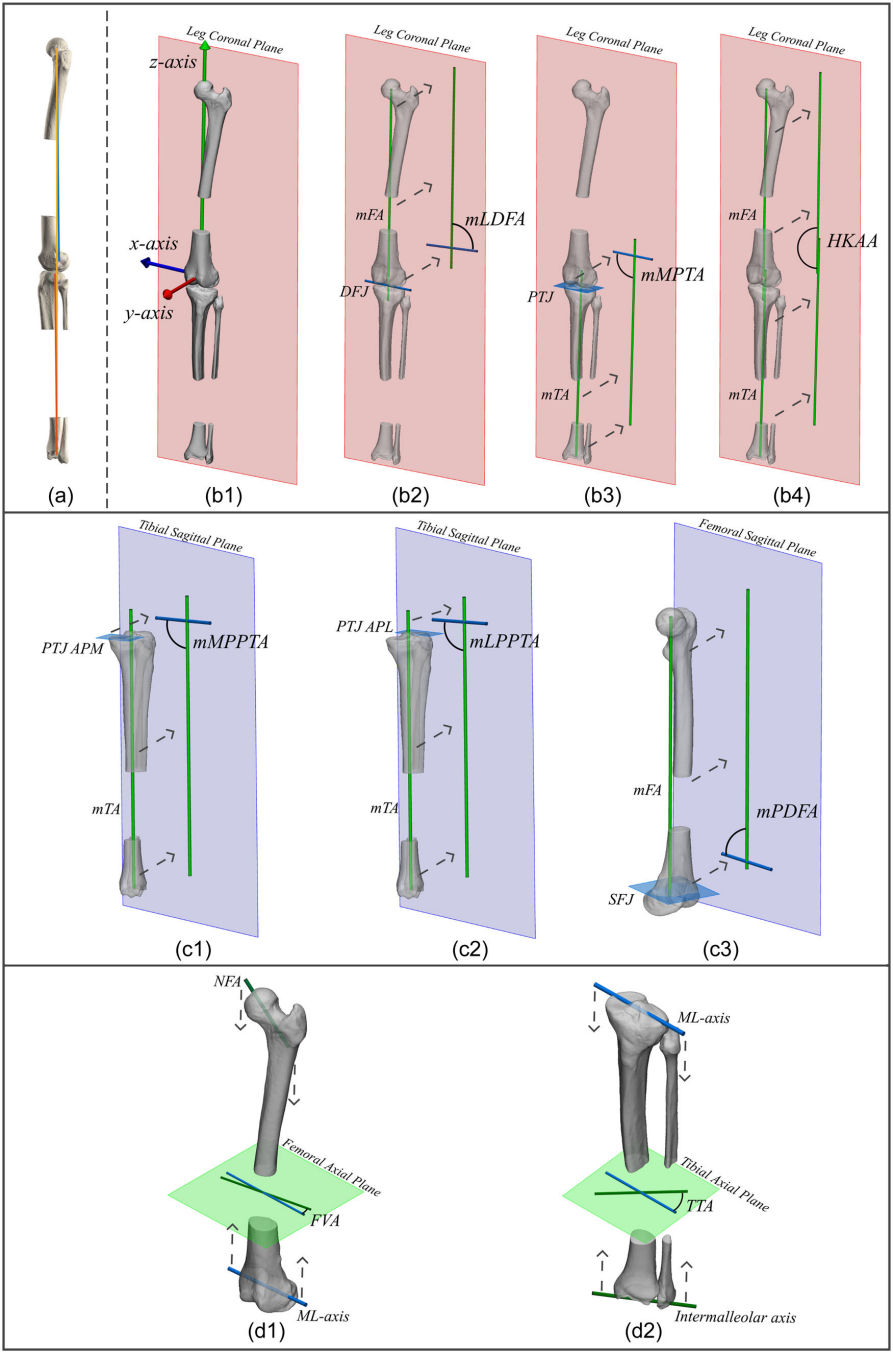
Illustrative example of clinical application of leg alignment analysis on 3D bone models based on the agreed upon principles in the present study: measuring leg alignment parameters in the anatomical plane of the corresponding coordinate system. The Figure demonstrates the integration of the agreed upon principles in the present study into a patient‐specific workflow. (a) To measure distal femoral and proximal tibial joint orientation angles and joint alignment in the coronal plane of the leg coordinate system, it is a prerequisite that the knee is extended. In a fully extended knee (i.e., mFA (blue) and mTA (red) are parallel in the sagittal plane of the leg coordinate system), the mFA and mTA are not necessarily in line with each other. Consequently, the mechanical leg axis (yellow) is not necessarily coincident with the mFA or mTA. (b) Measurement of distal femoral and proximal tibial joint orientation angles in the coronal plane of the leg coordinate system. (b1) The leg coronal plane results from the definition of the leg coordinate system. (b2) mLDFA: the lateral angle between the projected mFA (green) and DFJ (blue) on the leg coronal plane. (b3) mMPTA: the medial angle between the projection of the mTA (green) on, and the intersection of the PTJ (cyan plane) with the leg coronal plane. (b4) HKAA: the medial angle between the projection of the mTA (green, bottom) and mFA (green, top) on the leg coronal plane. (c) Measurement of distal femoral and proximal tibial joint orientation angles in the sagittal plane of their respective coordinate systems. (c1) mMPPTA: the posterior angle between the projection of the mTA (green) on, and intersection of the medial PTJ (cyan plane) with the tibial sagittal plane. (c2) mLPPTA: the posterior angle between the projection of the mTA (green) on, and the intersection of the lateral PTJ (cyan plane) with the tibial sagittal plane. (c3) mPDFA: the posterior angle of the projection of the mFA (green) on, and intersection of the SFJ (cyan plane) with the femoral sagittal plane. (d) Measurement of femoral version and tibial torsion. (d1) FVA: the acute angle between the projections of the NFA (green) and distal femoral central medial‐lateral joint axis (blue) on the femoral axial plane. (d2) TTA: the acute angle between the projections of the intermalleolar axis (green) and proximal tibial central medial‐lateral joint axis (blue) on the tibial axial plane. DFJ, distal femoral joint orientation; FVA, femoral version angle; HKAA, hip‐knee‐ankle angle; mFA, mechanical femoral axis; mLDFA, mechanical lateral distal femoral angle; mLPPTA, mechanical lateral posterior proximal tibial angle; mMPPTA, mechanical medial posterior proximal tibial angle; mMPTA, mechanical medial proximal tibial angle; ML, medio‐lateral; mPDFA, mechanical posterior distal femoral angle; mTA, mechanical tibial axis; NFA, neck‐femur axis; PTJ, proximal tibial joint orientation; PTJ APM, proximal tibial joint orientation anterior‐posterior medial; PTJ APL, proximal tibial joint orientation anterior‐posterior lateral; SFJ, supracondylar femoral joint orientation; TTA, tibial torsion angle.

With a framework for joint centres, femoral, tibial/fibular and leg coordinate systems, joint orientations, and proximal and distal medial‐lateral femoral and tibial/fibular axes, 3D leg alignment can be measured and expressed as alignment parameters in each anatomical reference plane of the relevant coordinate system (Figure 7). To this end, the expert panel agreed that joint alignment and distal femoral and proximal tibial coronal joint orientation angles should be calculated in the coronal plane of the leg coordinate system (Figure 7b), provided that the knee is extended (Figure 7a).

## DISCUSSION

The most important finding of this international Delphi consensus study is the agreement on a structured framework for standardized 3D leg alignment analysis using 3D bone models based on foundational principles. By defining these foundational principles, it addresses substantial methodological variability in existing literature, enhances clinical applicability for preoperative planning and execution of uni‐ and multiplanar correction osteotomies around the knee and total knee arthroplasty, and improves cross‐study comparability. This paves the way for more robust and standardized clinical and research applications in the field.

Despite significant advances in 3D technology and its growing adoption, many current ‘3D methods’ for deriving joint centres continue to rely on traditional 2D approaches, applied to 3D bone models [31]. These 3D methods often still use a specific anatomical landmark‐based approach, such as the anterior point of the notch to identify the distal femoral joint centre. While landmark‐based techniques have demonstrated high intra‐class correlation coefficients [13, 31], they are inherently limited in 3D context, since they cannot fully capture the joint's complex three‐dimensional geometry and thereby introduce potential inaccuracies. In contrast, the consensus approach leverages the full complexity of 3D bony anatomy, potentially reducing subjectivity and increasing reproducibility in alignment assessment.

While this study does not prescribe specific methods for defining the relevant available surface data, it establishes foundational principles to guide the development of a standardized and clinically applicable 3D leg alignment analysis framework. This consensus elevates the 3D framework to a comparable level of agreement as the 2D framework by providing agreed‐upon principles that ensure standardization while allowing flexibility in the applied method. Similar to 2D analyses – where multiple methods exist to determine key parameters (e.g., the distal femoral joint centre on a coronal plane radiograph: apex of the femoral notch, midpoint of femoral condyles, or centre of tibial spines [27]) – the 3D framework accommodates various methods to adhere to its principles, ensuring consistency despite methodological variability. These principles can be directly integrated into existing 3D‐based clinical workflows, enabling clinicians to optimize correction osteotomies based on patient‐specific anatomy. Future research should validate specific methods across diverse patient populations to ensure reliability and consistency in clinical practice.

For leg alignment analysis, a coordinate system is essential, as alignment parameters are expressed in anatomical planes of this system for purpose of reference. Axes of a coordinate system are often derived using landmarks such as the ‘most medial’ or ‘most lateral’ point on a 3D bone model [13, 31, 34], but these terms can only exist relative to a predefined reference coordinate system, creating circular reasoning. By leveraging all available relevant surface data from a 3D bone model, an axis can be obtained independently, without requiring an a priori reference system. This principle avoids reliance on single landmark points and addresses inherent redundancies, potentially providing a more robust foundation for deriving joint orientations and axes for 3D leg alignment analysis.

Joint alignment analysis (i.e., the collinearity of the joint centres of the hip, knee and ankle) is performed in the coronal plane of the leg [28]. However, a recent systematic review highlighted the absence of a well‐defined, complete leg coordinate system [31]. To address this, the expert panel established a leg coordinate system where the coronal plane is defined by the mechanical leg axis (Mikulicz line) and the medial‐lateral joint orientation axis of the distal femur, aligning closely with established clinical practices [28].

Although the leg coordinate system is designed independently of knee flexion, by definition, joint alignment analysis is standardized with the knee in extension [28]. Therefore, the panel agreed that coronal plane distal femoral and proximal tibial joint orientation angles, which significantly influence overall joint alignment, should also be calculated in the coronal plane of the leg coordinate system and with the knee in extension. This ensures that these crucial alignment parameters are consistently referenced within the leg coordinate system, rather than within individual femoral or tibial/fibular coordinate systems.

In cases where deformities prevent full knee extension, coronal plane joint orientation angles may be assessed within their respective femoral or tibial/fibular coordinate system, but such measurements might yield different values due to misalignment with the leg coordinate system. Virtual manipulation of 3D bone models into extension offers a significant advantage over the 2D framework, despite the complexity of this correction and potential for errors [5, 6]. Therefore, the authors propose to perform coronal plane joint alignment and joint orientation analysis in the coordinate system of the leg with the knee in extension, for which this consensus provides a robust framework.

Femoral version and tibial torsion are essential parameters for assessing rotational differences between the distal and proximal medial‐lateral joint orientation along a longitudinal axis [27]. The expert panel agreed that in 3D, version and torsion should be based on central medial‐lateral axes derived from all available relevant surface data.

Traditionally, even in 2D workflows, femoral version and tibial torsion are defined as the angle between the femoral neck and the intermalleolar axis, respectively, relative to the coronal plane [27, 34]. In this Delphi study, consensus was achieved that the coronal plane is dictated by the central medial‐lateral joint orientation axis, following the principle of utilizing all available relevant surface data. Consequently, femoral version and tibial torsion should be measured relative to this central medial‐lateral joint orientation, which adheres to the agreed upon principles in the present study.

This represents a shift from traditional 2D workflows, where these angles often rely on the posterior condylar axis as a medial‐lateral reference for the distal femur and proximal tibia. While practical on 2D CT‐slices, the posterior condylar axis is an indirect proxy that does not consistently align with the coronal plane defined by central axes. Importantly, a posterior condylar axis remains a valuable reference in 2D CT slices and surgical workflow for its simplicity, reproducibility and applicability [27]. To facilitate comparison between 2D and 3D methods, posterior condylar axis‐based measurements can be retained as supplementary alignment parameters. This consensus aims to establish fundamental definitions in 3D alignment analysis while allowing flexibility to incorporate additional alignment parameters that enhance clinical insights.

This consensus study provides a structured framework for analyzing 3D static anatomical alignment, particularly in the context of deformity analysis in a native leg for malalignment correction or total knee arthroplasty. However, for unilateral post‐traumatic or congenital deformities, it may be more effective to additionally use the bony morphology of the unaffected contralateral leg as a frame of reference, rather than relying solely on the femoral, tibial/fibular, and leg coordinate systems. For example, mirroring the unaffected side and overlaying it with the affected side could enable precise identification of the location and magnitude of deformities, providing a basis for planning malalignment corrections in such cases [2]. Nevertheless, reference coordinate systems, axes and joint orientations will still be required to calculate alignment parameters such as joint orientation angles and joint alignment.

Furthermore, current targets for leg alignment parameters rely on population‐based reference values, which serve as proxies for achieving optimal personalized joint loading. Future developments could build on this work by integrating functional data into malalignment correction planning and total knee arthroplasty [4, 35]. Such advancements would further refine the principles for 3D leg alignment analysis proposed in this study, enabling alignment corrections and total knee arthroplasty procedures to be more precisely tailored for optimizing joint mechanics during daily activities. This approach would not only complement the transition from 2D to 3D frameworks but also lay the groundwork for a patient‐specific 4D framework, incorporating 3D anatomical data with functional data to enhance clinical outcomes.

One methodological consideration in this study was the use of a binary agree/disagree scale rather than a Likert scale [7]. Given the high variability in methodologies for 3D leg alignment analysis [31], a binary scale was chosen to provide clear acceptance or rejection of statements while minimizing ambiguity. Unlike Likert scales, which allow for varying degrees of agreement but can introduce central tendency bias, the binary approach ensured a decisive consensus threshold while allowing qualitative refinement via free‐text comments. Previous research [21] has shown that simplified rating scales can yield similar consensus levels as more detailed scales in final decision‐making phases, supporting the robustness of our binary approach.

Despite achieving consensus, this study has several limitations. First, only two surveys were conducted, limiting the assessment of the statistical stability. Second, although efforts were made to balance international representation, European experts were overrepresented due to non‐response bias and existing professional networks. Although a systematic review found no clear regional differences in 3D leg alignment methods [31], broader global participation remains an area for improvement. Additionally, while a 50% response rate aligns with typical e‐Delphi studies and was sufficient to achieve meaningful consensus [7, 10, 14, 24], limited participation from certain regions highlights the need for more global inclusion in future research. Nonetheless, the expert panel maintained a balance between clinical and biomechanical expertise across multiple continents, ensuring both technical rigour and clinical relevance. Third, selection and response biases are inherent to Delphi studies due to the absence of universal criteria for panel composition [11, 33]. In this study, the rating group was selected from the steering group's professional network, and rating group members participated in the expert panel peer review group. While this approach may introduce selection bias, it reflects common Delphi methodology where expert networks are leveraged for recruitment. To enhance transparency, the selection and composition of each group were explicitly described. Additionally, the inclusion of participants with diverse backgrounds and international representation helped mitigate potential bias and ensured a balanced consensus. Fourth, while radiological software exists to facilitate the clinical implementation of the study's results, availability may vary. Finally, future research should focus on validating which available methods best align with the agreed‐upon principles in terms of reliability, feasibility, and clinical relevance.

## CONCLUSION

This international Delphi consensus study provides a structured framework for a standardized 3D leg alignment analysis, based on 3D bone models. This framework aims to enhance clinical applicability for preoperative planning and execution of uni‐ and multiplanar corrections osteotomies around the knee and total knee arthroplasty, reduce the current methodological variability in 3D leg alignment analysis literature, and improve cross‐study comparability.

## 3D Leg Alignment Consensus Expert Group

**Ahmet Erdemir** (Department of Biomedical Engineering, Lerner Research Institute, Cleveland Clinic, OH, USA, ORCID: 0000‐0002‐4629‐8055); **Antoine Perrier** (Laboratoire TIMC‐IMAG, University Grenoble Alpes, CNRS, Grenoble, France; Twinsight, Grenoble, France, ORCID: 0000‐0001‐8452‐0457); **Bastian Sigrist** (Center for 3D preoperative planning and 3D printing, University Hospital Balgrist, University of Zurich, Zurich, Switzerland, ORCID: 0000‐0002‐7720‐1420); **Bernardo Innocenti** (BEAMS Department, Université Libre de Bruxelles, Bruxelles, Belgium, ORCID: 0000‐0001‐8992‐8865); **Carl W. Imhauser** (Department of Biomechanics, Hospital for Special Surgery, New York, NY, USA, ORCID: 0000‐0003‐2445‐7112); **Claudio Belvedere** (Movement Analysis Laboratory, IRCCS Istituto Ortopedico Rizzoli, Bologna, Italy, ORCID: 0000‐0003‐4258‐2267); **Gwendolyn Vuurberg** (Department of Imaging, Radboud University Medical Centre, Nijmegen, the Netherlands, COI: 0000‐0001‐5008‐890X); **Harrie Weinans** (Department of Orthopaedics, University Medical Center Utrecht, Utrecht, the Netherlands, ORCID: 0000‐0002‐2275‐6170); **Julian Fürmetz** (Department of Trauma Surgery, BG Unfallklinik Murnau, Murnau, Germany; Department of Orthopaedics and Trauma Surgery, Musculoskeletal University Center Munich (MUM), University Hospital, LMU, Munich, Germany, ORCID: 0000‐0001‐5529‐1632); **Laura Carman** (Auckland Bioengineering Institute, the University of Auckland, New Zealand, ORCID: 0000‐0002‐2668‐7387); **Leendert Blankevoort** (Amsterdam UMC location University of Amsterdam, Department of Orthopaedic Surgery and Sports Medicine, Amsterdam, the Netherlands; Amsterdam Movement Sciences, Musculoskeletal Health, Amsterdam, the Netherlands, ORCID: 0000‐0002‐7810‐1659); **Mark Taylor** (Medical Device Research Institute, College of Science and Engineering, Flinders University, Adelaide, Australia, ORCID: 0000‐0001‐7842‐6472); **Mathias Donnez** (Newclip Technics, Haute‐Goulaine, France, ORCID: 0000‐0002‐4595‐4057); **Matthias J. Feucht** (Department of Orthopedic Surgery and Traumatology, Freiburg University Hospital, Albert Ludwigs University Freiburg, Freiburg, Germany, ORCID: 0000‐0002‐7639‐9105); **Matthieu Ollivier** (Aix Marseille Univ, CNRS, ISM, Marseille, France, ORCID: 0009‐0009‐7025‐2776); **Michael T. Hirschmann** (Department of Orthopaedic Surgery and Traumatology, Kantonsspital Baselland, Bruderholz, Switzerland; Department of Clinical Research, Research Group Michael T. Hirschmann, Regenerative Medicine & Biomechanics, University of Basel, Basel, Switzerland, ORCID: 0000‐0002‐4014‐424X); **Min Jung** (Department of Orthopaedic Surgery, Arthroscopy and Joint Research Institute, Yonsei University College of Medicine, Seoul, Republic of Korea, ORCID: 0000‐0002‐7527‐4802); **Oguzhan Tanoğlu** (Department of Orthopaedics and Traumatology, İzmir Democracy University, İzmir, Turkey, ORCID: 0000‐0001‐8984‐9008); **Philipp Niemeyer** (OCM, Orthopädische Chirurgie München, Munich, Germany, ORCID: 0000‐0001‐6271‐3733); **Raghbir Khakha** (Department of Trauma and Orthopaedics, Guys and St Thomas Hospital, London, United Kingdom, ORCID: 0000‐0003‐0705‐3896); **Roel J.H. Custers** (Department of Orthopaedics, University Medical Center Utrecht, Utrecht, the Netherlands, ORCID: 0000‐0001‐5253‐2883); **Ronald van Heerwaarden** (Centre for Deformity Correction and Joint Preserving Surgery, Kliniek ViaSana, Mill, the Netherlands, ORCID: 0000‐0003‐2686‐5830); **Ruurd J.A. Kuiper** (Department of Orthopaedics, University Medical Center Utrecht, Utrecht, the Netherlands; Image Sciences Institute, University Medical Center Utrecht, Utrecht, the Netherlands, ORCID: 0000‐0002‐6511‐3896); **Sandro F. Fucentese** (Department of Orthopaedics and Traumatology, University Hospital Balgrist, University of Zurich, Zurich, Switzerland, ORCID: 0000‐0003‐2993‐7537); **Steven Claes** (Orthopedic Department, AZ Herentals Hospital, Antwerp, Belgium); **Thor F. Besier** (Auckland Bioengineering Institute, University of Auckland, New Zealand; Department of Engineering Science & Biomedical Engineering, University of Auckland, New Zealand, ORCID: 0000‐0003‐0818‐7554); **Vicente J. León‐Muñoz** (Instituto de Cirugía Avanzada de la Rodilla (ICAR), Murcia, Spain; Department of Surgery, Paediatrics and Obstetrics & Gynaecology (Faculty of Medicine; Murcia University), Murcia, Spain; Department of Orthopaedic Surgery and Traumatology, Hospital General Universitario Reina Sofía, Murcia, Spain, ORCID: 0000‐0002‐0429‐2579); **Wolf Petersen** (Klinik für Orthopädie und Unfallchirurgie, Martin Luther Hospital, Berlin, Germany, ORCID: 0000‐0002‐6834‐4438); **Wouter van Genechten** (Orthopaedic department, University hospital Antwerp, Antwerp, Belgium, ORCID: 0000‐0001‐5844‐9247); **Yuanjun Teng** (Academy for Engineering and Technology, Fudan University, Shanghai, China; Department of Orthopaedic Surgery, Huashan Hospital, Fudan University, Shanghai, China, ORCID: 0000‐0002‐3163‐954X).

## AUTHOR CONTRIBUTIONS

Quinten W.T. Veerman, Gabriëlle J.M. Tuijthof, Nico Verdonschot, Reinoud W. Brouwer, Peter Verdonk, Annemieke van Haver, Hugo C. van der Veen, Peter Pijpker, Judith olde Heuvel and Roy A.G. Hoogeslag contributed to the study conception, design and initial development of statements. All authors contributed to the investigation and data generation. The first draft of the manuscript was written and edited by Quinten W.T. Veerman, Gabriëlle J.M. Tuijthof, Nico Verdonschot, Judith olde Heuvel and Roy A.G. Hoogeslag. The final version of the manuscript was reviewed and edited by Ahmet Erdemir, Annemieke van Haver, Bastian Sigrist, Bernardo Innocenti, Carl Imhauser, Gwendolyn Vuurberg, Harrie Weinans, Hugo C. van der Veen, Julian Fürmetz, Leendert Blankevoort, Peter Verdonk, Reinoud W. Brouwer, Ruurd J.A. Kuiper, Sandro F. Fucentese and Vicente J. León‐Muñoz. The research was supervised by Gabriëlle J.M. Tuijthof, Nico Verdonschot, Judith olde Heuvel and Roy A.G. Hoogeslag. All authors read and approved the final manuscript.

## CONFLICT OF INTEREST STATEMENT

Matthieu Ollivier is a consultant and receives royalties from Newclip Technics and Stryker. Sandro F. Fucentese is a paid consultant at Medacta, Zimmer Biomet and Karl Storz; AJSM reviewer and EKA‐ESSKA Osteotomy Board Member; has given paid presentations on Medacta and S&N products in the last 12 months. Wolf Petersen receives consulting fees from Arthrex (Munich), AAP implants (Berlin), and OPED (Valley, Germany). None of the other authors have declared any conflicts of interest. Additionally, M.T. Hirschmann and M. Ollivier are editorial board members of KSSTA.

## ETHICS STATEMENT

Ethical approval was not required for this Delphi study.

## Supporting information

Supporting information.

## Data Availability

All data is made anonymously available in the Supporting S1 Information.
