# Supplementary material for: A structured framework for standardized 3D leg alignment analysis: An international Delphi consensus study
Source: Knee Surg Sports Traumatol Arthrosc. 2025 Apr 16;33(6):2276–92. doi: 10.1002/ksa.12676 (PMC12104791; doi:10.1002/ksa.12676)
Supplement: Supplementary file 1 — Supporting information. [file KSA-33-2276-s001.pdf]

SUPPLEMENTARY INFORMATION

Supplementary information to:

Title A structured framework for standardized 3D leg alignment analysis: an international Delphi consensus study

Journal KSSTA

Authors Quinten W.T. Veerman, Gabriëlle J.M. Tuijthof, Nico Verdonschot, Reinoud W. Brouwer, Peter Verdonk, Annemieke van Haver, Hugo C. van der Veen, Peter A.J. Pijpker, Judith olde Heuvel, Roy A.G. Hoogeslag, and the *3D Leg Alignment Consensus Expert Group*.

3D Leg Alignment Consensus Expert Group
Ahmet Erdemir ; Antoine Perrier ; Bastian Sigrist; Bernardo Innocenti ; Carl Imhauser ; Claudio Belvedere ; Gwendolyn Vuurberg ; Harrie Weinans ; Julian Fürmetz ; Laura Carman ; Leendert Blankevoort ; Mark Taylor ; Mathias Donnez ; Matthias J. Feucht ; Matthieu Ollivier ; Michael T. Hirschmann ; Min Jung ; Oguzhan Tanoğlu ; Philipp Niemeyer ; Raghbir Khakha ; Roel Custers ; Ronald van Heerwaarden ; Ruurd J.A. Kuiper ; Sandro F. Fucentese ; Steven Claes ; Thor F. Besier ; Vicente J. León-Muñoz ; Wolf Petersen ; Wouter van Genechten ; Yuanjun Teng

Corresponding author Quinten W.T. Veerman; q.veerman@ocon.nl

A. Surveys

A1. Round 3 complete survey

<u>GENERAL INTRODUCTION AND PURPOSE OF THE STUDY</u>

We would like to thank you for joining us in a Delphi survey on the principles on how to derive axes and joint orientations from 3D bone models of the lower extremity.

Rationale

In osteotomies around the knee and total knee arthroplasty, 3D bone models are increasingly being used for leg alignment analysis. However, in a recent systematic review [1] the authors found that there is a high variability in the methods on how to derive axes and joint orientations from 3D models that are necessary for the definition of leg alignment parameters and coordinate system in which anatomical reference planes leg alignment parameters are expressed.

The variability in methods to derive axes and joint orientations from 3D bone models to define alignment parameters could lead to different alignment parameters values between studies, which might render the comparability between studies impossible. It implies that the ‘normal values’ for alignment parameters in 3D models reported in several studies are only valid within the context of those studies and cannot be broadly adopted. This might lead to inappropriate malalignment correction or total knee arthroplasty placement.

Therefore, consensus on a 3D framework seems essential.

Delphi method

As you might know, a Delphi survey is a scientific iterative method to structure expert discussion and aim to find consensus where possible. The survey was already tested by the working group, and will be sent to you twice. This first time the survey will include all statements. After analysis of the received answers and remarks, we will send you the survey a second time, with the remaining statements not yet meeting consensus.

It is important to mention that your responses will always be reported anonymously.

What we ask from you

For each subject, you are asked about principles on how to derive axes and joint orientations from 3D bone models of the lower extremity to establish knee-related alignment parameters and coordinate systems. For illustration purposes of the proposed principles, one of many methods to use this proposed principle is included with every question, such as calculation of centroids or fitting geometrical shapes to all relevant 3D model’s surface data. It is important to note that the goal is **not** to discuss the methods of the illustration, but merely to discuss the underlying principle. If you do not agree, you are encouraged to explain why you disagree in the optional “comments / remarks” section.

The survey contains 31 agree/disagree questions, and is expected to take about 25-30 minutes.

3D leg alignment analysis working group

Quinten W.T. Veerman, Gabriëlle J.M. Tuijthof, Nico Verdonschot, Reinoud W. Brouwer, Peter Verdonk, Annemieke van Haver, Hugo C. van der Veen, Peter A.J. Pijpker, Judith olde Heuvel, Roy A.G. Hoogeslag

----

[1] Veerman, Q. W. T., ten Heggeler, R. M., Tuijthof, G. J. M., Graaff, F., Fluit, R. & Hoogeslag, R. A. G.

(2024) High variability exists in 3D leg alignment analysis, but underlying principles that might lead to agreement on a universal framework could be identified: A systematic review. Knee Surgery, Sports Traumatology, Arthroscopy, 1–15. https://doi.org/10.1002/ksa.12512

START OF THE SURVEY

This survey consists of 5 parts, each with a specific focus.

1. Joint centers: In the first part, principles on how to derive joint centers from a 3D bone model are proposed. With this information, mechanical axes of the femur, tibia/fibula, and leg can be established.

2. Joint orientations: In the second part, principles on how to derive distal femoral and proximal tibial 3D joint orientations from the respective 3D bone models are proposed. With this information, together with the mechanical axes, 3D joint orientation angles for the knee can be established.

3. Individual femoral and tibial/fibular coordinate systems: In the third part, principles on how to derive femoral and tibial/fibular coordinate systems from the respective 3D bone models are proposed. With this information, 3D joint orientations and axes can be projected on femoral and tibial/fibular anatomical reference planes so that they can be expressed as joint orientation angles that are relevant for knee-related leg alignment analysis.

4. Combined femoral and tibial/fibular (leg) coordinate system: In the fourth part, principles on how to derive the leg coordinate system from the combined femoral and tibial/fibular 3D bone models are proposed. With this information, 3D joint orientations and axes can be projected on the leg coronal anatomical reference plane so that they can be expressed as joint alignment and coronal plane joint orientation angles that are relevant for knee-related leg alignment analysis.

5. Femoral version and tibial torsion: In the fifth and last part, principles on how to derive proximal and distal medial-lateral femoral and tibial joint orientations from their respective 3D bone models are proposed. With this information, distal and proximal medial-lateral joint orientations can be projected on axial anatomical reference planes of their respective coordinate system so that they can be expressed as femoral version or tibial torsion.

Furthermore, in the following survey, the principle to use all relevant data of a 3D bone model to derive axes and joint orientation from a 3D bone model is used. While 3D bone models offer a plethora of bony surface data for defining 3D joint orientation and joint alignment, methods for the derivation of axes and joint orientation from 3D bone models span a wide spectrum: from the use of an arbitrary single landmark point to using all available relevant surface data of a 3D bone model (see figure below). While the positioning of single landmark points on 3D bone models has shown high reliability, this is merely an application of 2D principles on 3D bone models, neglecting a vast amount of the available information of the 3D bone morphology. Instead of using single points, employing all relevant surface data of a 3D bone model seems more robust, and could allow for more reproducible 3D alignment analysis.

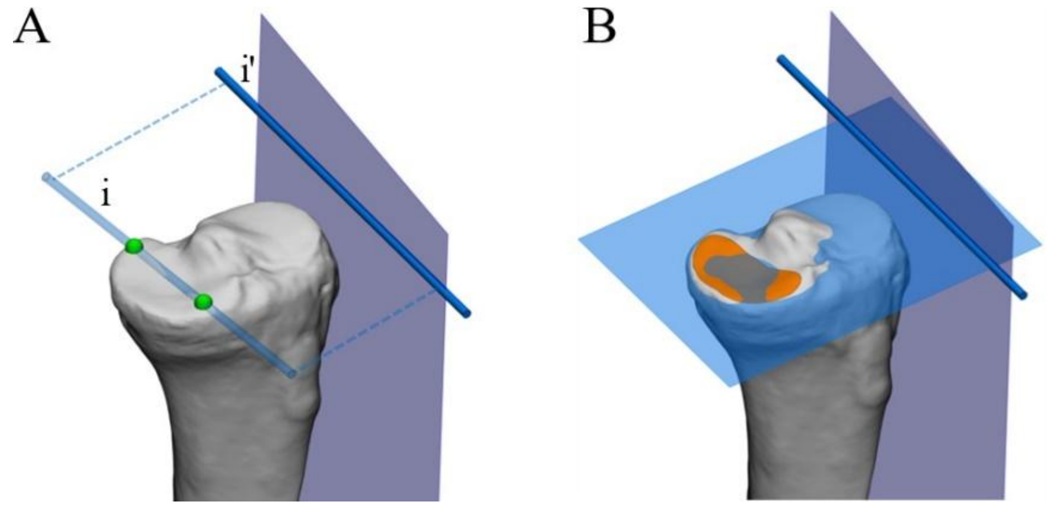

Figure 1 Example of the disparity between methods to obtain the 3D joint orientation of the medial tibial plateau. A: a line (i; blue; translucent) between two landmark points (green) defines the 2D anterior-posterior medial tibial plateau joint orientation, one point on the anterior and one point on the posterior border of the medial tibial plateau; the projection of this line on the coordinate system's sagittal plane (purple) defines the medial tibial plateau's sagittal joint orientation (i'). B: all surface data of the medial tibial plateau's articular surface (orange) define the 3D medial tibial plateau's joint orientation (light blue plane); the intersecting line of the medial tibial plateau plane with the coordinate system's sagittal plane (purple) defines the medial tibial plateau's sagittal joint orientation (blue line).

QUESTIONNAIRE TO PART 1 OF 5 OF THE SURVEY

Joint centers

In part one of this survey, principles on how to derive joint centers from a 3D bone model are proposed. With this information, mechanical axes of the femur, tibia/fibula, and leg can be established.

Proximal Femoral Joint Center

1. The proximal femoral joint center is derived from all available surface data of the articular surface of the femoral head (Figure 2).

   AGREE / DO NOT AGREE

   REMARKS:

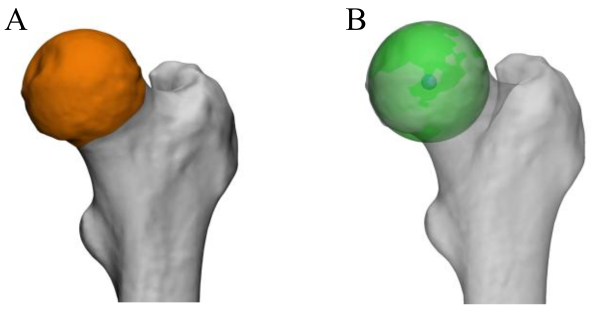

Figure 1: Possible method to obtain the proximal femoral joint center derived from all articular surface data of the femoral head. A: proximal femur with identification of the articular surface of the femoral head (orange). B: a best fit sphere (green) could be fit to the articular surface of the femoral head (marked in 2A) and the resulting centroid (blue) could be used as proximal femoral joint center.

Distal Femoral Joint Center

2. The articular surface of the distal femur is divided into three segments (Figure 3):
   a. the medial femoral condyle
   b. the lateral femoral condyle
   c. and the trochlea

   The femoral condyles and trochlea are divided at the level of the linea terminalis, and the medial and lateral femoral condyles are divided at the level of the deepest point of the trochlea and/or the highest point of the notch (Figure 3).

   AGREE / DO NOT AGREE

   REMARKS:

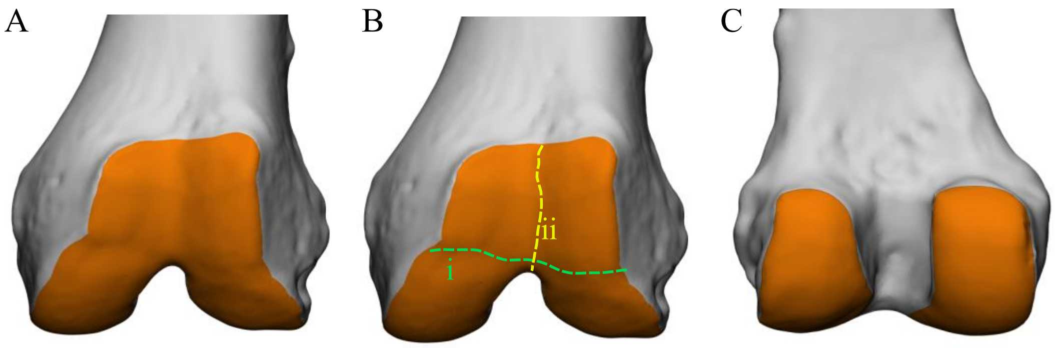

Figure 2: Possible method to divide the distal femur into the medial condyle, lateral condyle and trochlea based on all articular surface data of the distal femur. A: distal femur with identification of the articular surface of the trochlea and medial and lateral femoral condyles (orange). B: the linea terminalis (i; green dashed) separating the condyles from the trochlea, and trochlear groove line (ii; yellow dashed) separating the medial from the lateral condyle. C: posterior-anterior view of the distal femur with the medial and lateral articular surfaces (orange).

3. The distal femoral joint center is derived from all available surface data of the articular surface of the medial and lateral distal femoral condyles (Figure 4).

   AGREE / DO NOT AGREE

   REMARKS:

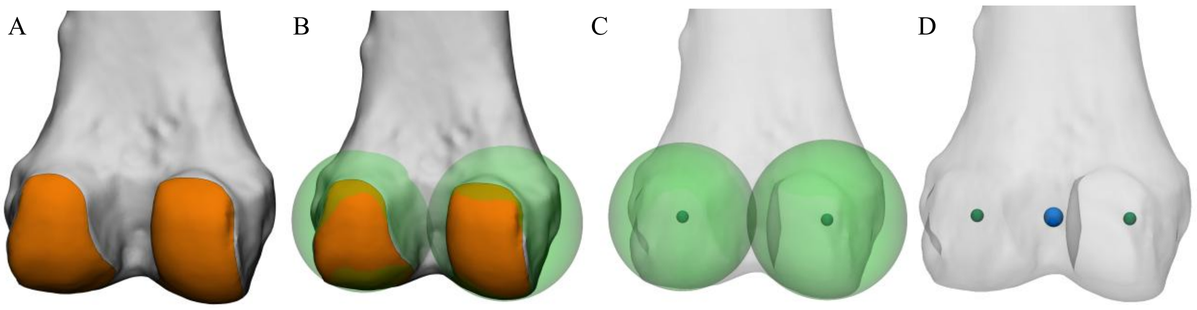

Figure 3: Possible method to obtain the distal femoral joint center derived from all articular surface data of the femoral head. A: posterior-anterior view of distal femur with identification of the articular surfaces of the medial and lateral condyles (orange). B: best fit spheres could be fit to the articular surface of each femoral condyle (green). C: identification of the resulting centroids of the best fit spheres to the femoral condyles (dark green). D: the midpoint of the centroids to the best fit spheres could be used to define the distal femoral joint center (blue).

Proximal Tibial/Fibular Joint Center

4. The proximal tibial joint center is derived from all available surface data of the articular surface of the medial and lateral tibial plateau (Figure 5).

   AGREE / DO NOT AGREE

   REMARKS:

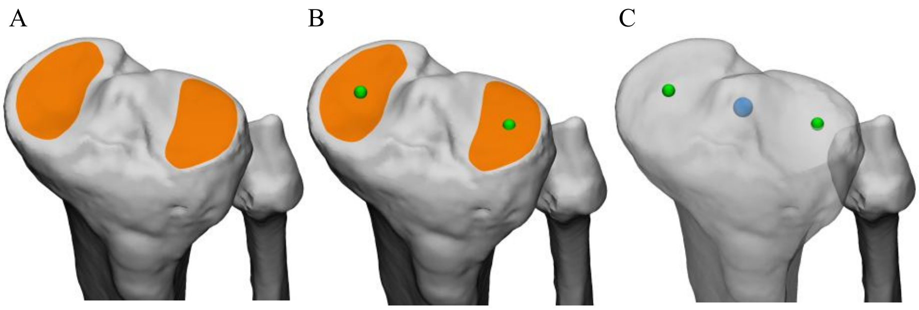

Figure 4: Possible method to obtain the proximal tibial joint center. A: identification of the articular surfaces of the medial and lateral tibial plateau (orange). B: identification of the resulting centroids of each of the articular surfaces of the medial and lateral tibial plateau (green). C: the midpoint of the centroids could be used to define the proximal tibial joint center (blue).

Distal Tibial/Fibular Joint Center

5. The articular surface of the distal tibial/fibular is divided into three segments (Figure 6):
    a. the medial malleolus
    b. the lateral malleolus
    c. and the tibial plafond

AGREE / DO NOT AGREE

REMARKS:

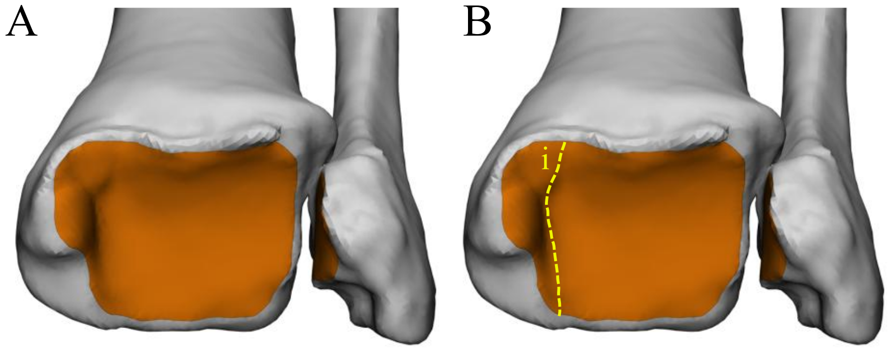

Figure 5: Possible method of how to divide the distal tibial articular surface of the medial malleolus and the tibial plafond based on all articular surface data of the distal tibia. A: distal tibia and fibula with identification of the articular surface of the tibial plafond and medial and lateral malleolus (orange). B: the medial gutter (i; yellow dashed) could separate the medial malleolus from the tibial plafond articular surface.

6. The distal tibial/fibular joint center is derived from all available surface data of the combined distal tibial and fibular articular surfaces (Figure 7)

AGREE / DO NOT AGREE

REMARKS:

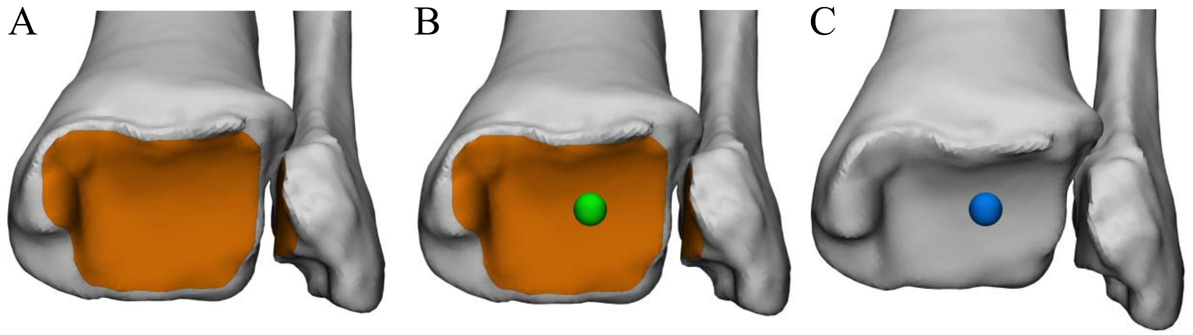

Figure 6: Possible method to obtain the distal tibial/fibular joint center, anterior-posterior view. A: distal tibia and fibula with identification of the articular surface of the tibial plafond and medial and lateral malleolus (orange). B: identification of the resulting centroid from the distal tibial/fibular articular surfaces (green). C: the centroid could be used to define the distal tibial/fibular joint center (blue).

Mechanical axes

Now that the principles on how to derive the femoral and tibial/fibular joint centers from a 3D bone model are defined, the mechanical axes can be established. The femoral mechanical axis is the line between the proximal and distal femoral joint centers. The tibial/fibular mechanical axis is the line between the proximal and distal tibial/fibular joint centers. And the mechanical leg axis (Mikulicz line) is the line between the proximal femoral and the distal tibial/fibular joint centers.

QUESTIONNAIRE TO PART 2 OF 5 OF THE SURVEY

Joint orientations

In part two of this survey, principles on how to derive distal femoral and proximal tibial 3D joint orientations from a 3D bone model are proposed. With this information, together with the mechanical axes, 3D joint orientation angles for the knee can be established.

Distal Femoral Joint Orientation

7. The distal femoral condylar joint orientation is based on the most distal point(s) (relative to a reference coordinate system; part 3 of the survey) of the articular surfaces of the medial and lateral femoral condyle (Figure 8).

   AGREE / DO NOT AGREE

   REMARKS:

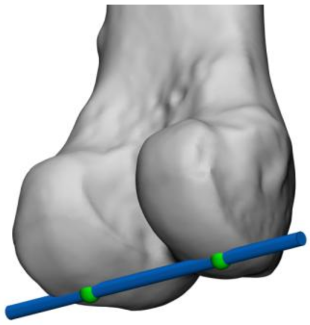

Figure 8: Possible method of how to obtain the distal femoral condylar joint orientation. A tangent line (blue) could be obtained that connects the most distal points (green) relative to a reference coordinate system of all available surface data of the medial and lateral femoral condyle articular surfaces.

8. The femoral supracondylar-trochlear orientation is based on the most proximal borders (relative to a reference coordinate system; part 3 of the survey) of all available surface data of the articular surfaces of the trochlea and medial and lateral femoral condyles (Figure 9).

   AGREE / DO NOT AGREE

   REMARKS:

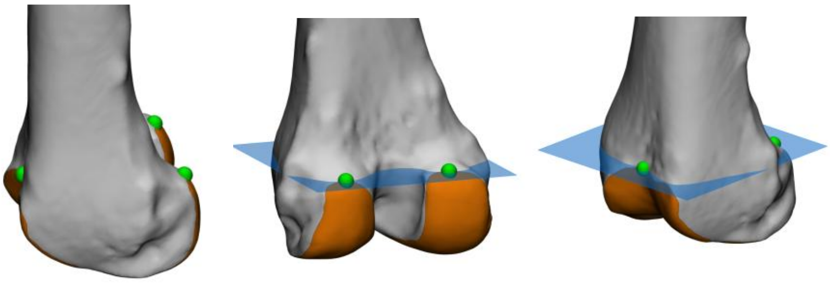

Figure 9: Possible method of how to obtain the femoral supracondylar-trochlear orientation. A plane (blue) could be obtained using the most proximal points (green; relative to a reference coordinate system) of all available surface data of the articular surfaces (orange) of the trochlea and medial and lateral femoral condyles.

Proximal Tibial Joint Orientation

9. The medial tibial plateau's joint orientation is derived from all available surface data of the medial tibial plateau's articular surface (Figure 10).

   AGREE / DO NOT AGREE

   REMARKS:

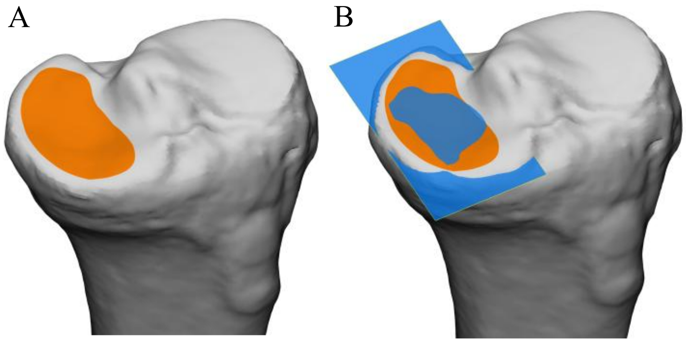

Figure 10: Possible method of how to obtain the medial tibial plateau's joint orientation. A: identification of the articular surface of the medial tibia plateau (orange). B: a plane (blue) could be fit through the articular surface.

10. The lateral tibial plateau's joint orientation is derived from all available surface data of the lateral tibial plateau's articular surface (Figure 11).

    AGREE / DO NOT AGREE

    REMARKS:

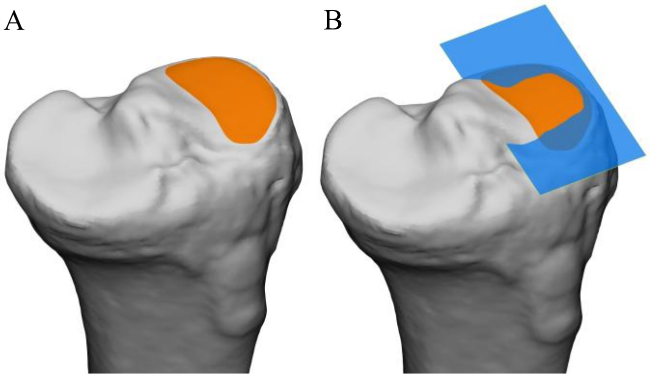

Figure 11: Possible method of how to obtain the lateral tibial plateau's joint orientation. A: identification of the articular surface of the lateral tibial plateau (orange). B: a plane (blue) could be fit through the articular surface.

11. The tibial plateau's joint orientation is derived from all available surface data of the medial and lateral tibial plateau's articular surface (Figure 12).

    AGREE / DO NOT AGREE

    REMARKS:

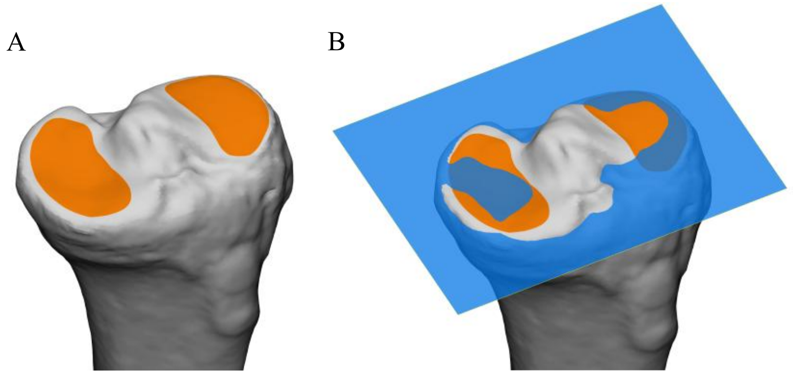

Figure 12: Possible method of how to obtain the tibial plateau's joint orientation. A: identification of the articular surfaces of the medial and lateral tibial plateaus (orange). B: a plane (blue) could be fit through the articular surfaces.

QUESTIONNAIRE TO PART 3 OF 5 OF THE SURVEY

Individual femoral and tibial/fibular coordinate systems

After determining axes and joint orientations, joint alignment and joint orientation angles can be analyzed. Although these parameters inherently exist in 3D space, for purpose of reference, they are projected onto the coronal, sagittal, or axial anatomical planes. Consequently, it becomes essential to precisely and reproducibly define the relevant coordinate systems and anatomical reference planes.

The gold standard to establish 3D joint coordinate systems and anatomical reference planes stems from the International Society of Biomechanics' "recommendation on definition of coordinate systems" [3]. The femoral coordinate system is based on the medial-lateral direction of the distal femur and the mechanical femoral axis, while the tibial/fibular coordinate system is based on the medial-lateral direction of the tibial plateau and the mechanical tibial axis. Both the mechanical axes and the medial-lateral direction of the distal femur and proximal tibia/fibula are clinically relevant terms, especially in the context of mechanical leg alignment analysis [1].

Of contention is the ISB's reliance on single datapoint landmarks only (similar to the 2D alignment analysis method), while in 3D bone models there is access to a multitude of points in the available surface data. Furthermore, the ISB suggests these landmarks as arbitrary points like 'most medial', 'most lateral' and 'most distal'. This, however, implies a pre-existent reference system, which is not the case, leading to circular reasoning. A more robust and independent approach to define a coordinate system from the ground up should harness all available relevant surface data from the 3D bone model.

Moreover, the ISB suggests the hip joint center and the ankle joint center serve as origins for the femoral and tibial/fibular coordinate systems, respectively. However, for a knee-focused coordinate system, the distal femoral joint center and the proximal tibial joint center could be viable alternatives.

In part three of this survey, principles on how to derive axes and origins from a 3D bone model to derive coordinate systems of the femur and tibia/fibula are proposed. With this information, 3D joint orientations and axes can be projected on femoral and tibial/fibular anatomical reference planes so that they can be expressed as joint orientation angles that are relevant for knee-related leg alignment analysis.

Coordinate system nomenclature and direction of axes

12. Conform the ISB recommendations, the distal-proximal axis is the y-axis, and points cranially; the medial-lateral axis is the z-axis, and points laterally; and the posterior-anterior axis is the x-axis, and points anteriorly.

    AGREE / DO NOT AGREE

    REMARKS:

Femoral coordinate system

13. The femoral coordinate system's origin is coincident with the distal femoral joint center (Figure 14).

    AGREE / DO NOT AGREE

    REMARKS:

14. The femoral coordinate system's distal-proximal axis (y-axis) is parallel with the mechanical femoral axis. (Figure 14).

AGREE / DO NOT AGREE

REMARKS:

15. The femoral coordinate system's medial-lateral axis (z-axis) is parallel to the projection (in the direction of the mechanical femoral axis) of the medial-lateral axis of the distal femur on the femoral axial plane. (Figure 14).

AGREE / DO NOT AGREE

REMARKS:

16. The femoral coordinate system's posterior-anterior axis (x-axis) is orthogonal to the y- and z-axis. (Figure 14).

AGREE / DO NOT AGREE

REMARKS:

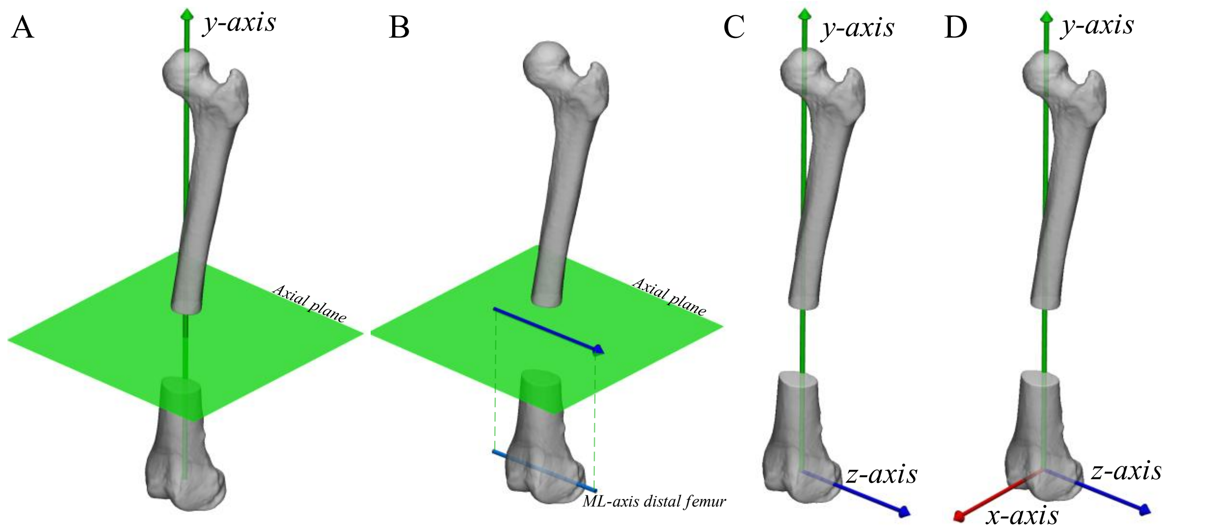

Figure 14: Femoral coordinate system. A: the direction of the proximal-distal axis (y-axis; green arrow) is defined parallel to the mechanical femoral axis, thereby also defining the axial plane (green plane). B: the direction of the medial-lateral axis (z-axis; blue arrow) is defined parallel to the medial-lateral axis of the distal femur (cyan cylinder), projected on the axial plane in the direction of the mechanical femoral axis (green dashed). C: resulting y- and z-axis defined from the origin (distal femoral joint center). D: the anterior-posterior axis (x-axis; red arrow) follows from being orthogonal to both the y- and z-axis. Abbreviations: ML, medial-lateral.

17. For the femoral coordinate system, the medial-lateral z-axis of the distal femur is derived from all available surface data of the articular surface of the medial and lateral distal femoral condyles (Figure 15).

AGREE / DO NOT AGREE

REMARKS:

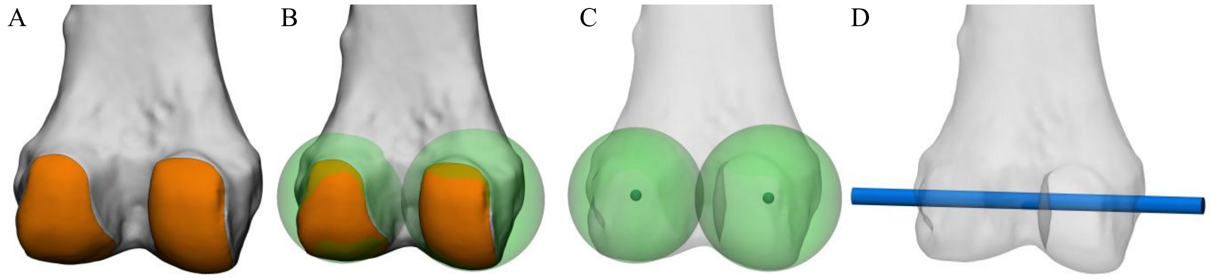

Figure 15: Possible method of how to obtain the medial-lateral axis of the distal femur, posterior-anterior view. A: articular surfaces of the medial and lateral condyles (orange). B: best fit spheres to each articular surface (green). C: resulting centroids from the fit spheres (dark green). D: the medial-lateral axis of the distal femur could be obtained by connecting the centroids.

Tibial/fibular coordinate system

18. The tibial/fibular coordinate system's origin is coincident with the proximal tibial joint center (Figure 16).

    AGREE / DO NOT AGREE

    REMARKS:

19. The tibial/fibular coordinate system's distal-proximal axis (y-axis) is parallel to the mechanical tibial axis (Figure 16).

    AGREE / DO NOT AGREE

    REMARKS:

20. The tibial/fibular coordinate system's medial-lateral axis (z-axis) is parallel to the projection (in the direction of the mechanical tibial axis) of the medial-lateral axis of the proximal tibia on the tibial axial plane (Figure 16).

    AGREE / DO NOT AGREE

    REMARKS:

21. The tibial/fibular coordinate system's posterior-anterior axis (x-axis) is orthogonal to the y- and z-axis (Figure 16).

AGREE / DO NOT AGREE

REMARKS:

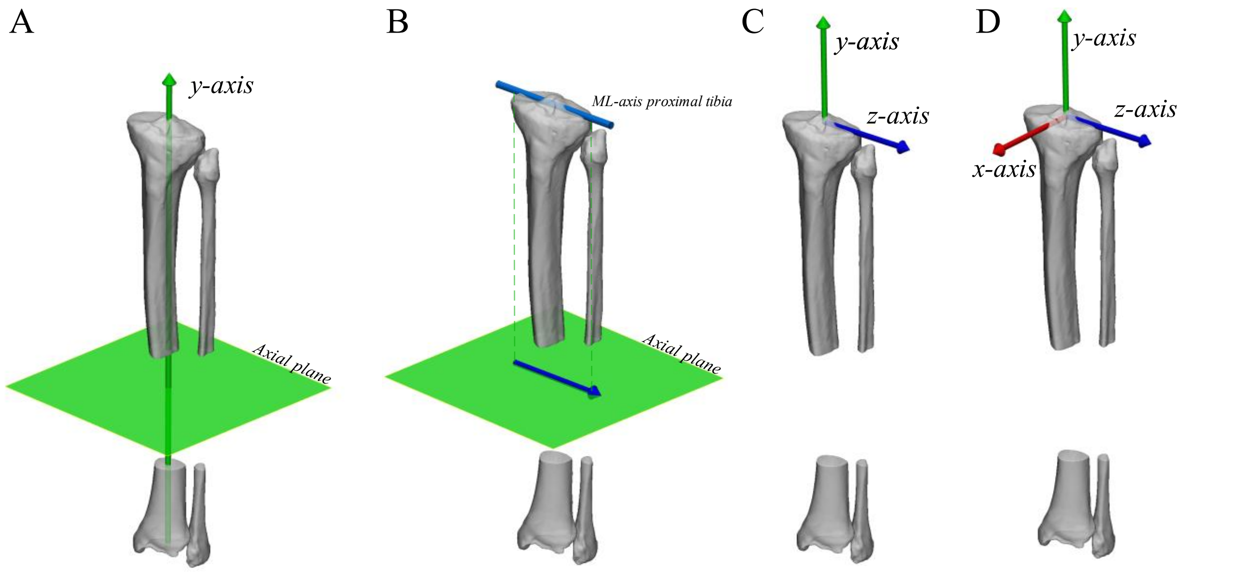

Figure 16: Tibial coordinate system. A: the direction of the proximal-distal axis (y-axis; green arrow) is defined parallel to the mechanical tibial axis, thereby also defining the axial plane (green plane). B: the direction of the medial-lateral axis (z-axis; blue arrow) is defined parallel to the medial-lateral axis of the proximal tibia (cyan cylinder), projected on the axial plane in the direction of the mechanical tibial axis (green dashed). C: resulting y- and z-axis defined from the origin (proximal tibial joint center). D: the posterior-anterior axis (x-axis; red arrow) follows from being orthogonal to both the y- and z-axis. Abbreviations: ML, medial-lateral.

22. For the tibial coordinate system, the medial-lateral z-axis is derived from all available surface data of the articular surface of the medial and lateral tibial plateau (Figure 17).

    AGREE / DO NOT AGREE

    REMARKS:

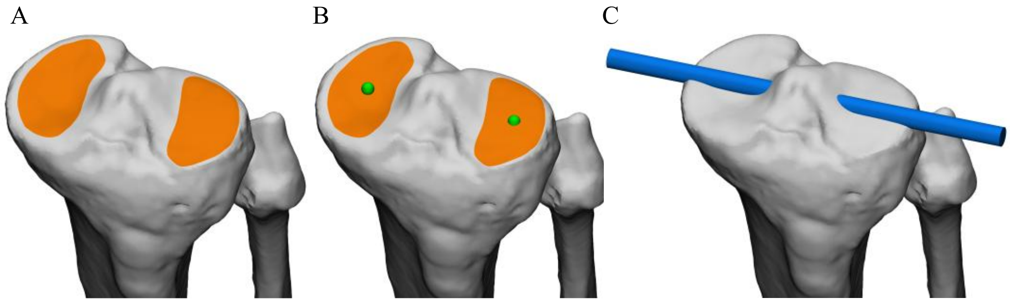

Figure 17: Possible method of how to obtain the medial-lateral axis of the proximal tibia, anterolateral view. A: articular surfaces of the medial and lateral tibial plateau (orange). B: resulting centroids of each articular surface (green). C: the medial-lateral axis of the proximal tibia (blue) could be obtained by connecting the centroids.

QUESTIONNAIRE TO PART 4 OF 5 OF THE SURVEY

Leg coordinate system

Although the ISB does recommend on the local 3D joint coordinate systems of the femur and tibia/fibula, it lacks a definition of a local leg coordinate system [3]. However, in 2D, joint alignment and coronal plane joint orientation analysis are performed in the coronal plane of the leg, on a long leg X-ray. The guideline that dictates the technique for a long leg X-ray inherently defines the leg's coronal plane, and therefore the leg coordinate system [2]. Here, the X-ray beam is positioned parallel, and its detector orthogonal, to the ground, and the patient stands weight-bearing with bipedal stance and extended knees [2]. This supports that the leg's coronal plane aligns with the mechanical leg axis. And although knee extension implicates that the mechanical axes of the femur and tibia are parallel (in the sagittal plane of the leg coordinate system), they are not necessarily in line with each other, but can be translated ad latum. Therefore, the mechanical axis of the leg is not parallel to the femoral or tibial mechanical axes per se (Figure 18). Furthermore, the patient stands with the 'knee-forward plane' [2] or the 'plane through the flexion-extension axes of the distal femur' parallel to the X-ray detector [2], supporting that the leg's coronal plane aligns with the medial-lateral direction of the distal femur. Thus, joint alignment and coronal plane joint orientation angles are defined in the coronal plane of the leg's coordinate system, which is dictated by the mechanical leg axis and the medial-lateral direction of the distal femur, with the prerequisite that the knee is extended.

In part four of this survey, principles on how to define axes and an origin from a 3D bone model to define the leg coordinate system are proposed. With this information, 3D joint orientations and axes can be projected on the leg coronal anatomical reference plane so that they can be expressed as joint alignment and coronal plane joint orientation angles that are relevant for knee-related alignment analysis.

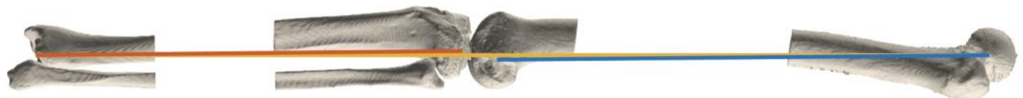

Figure 18: In a fully extended knee (i.e., mechanical femoral axis (mFA; blue) and mechanical tibial axis (mTA; red) are parallel in the sagittal plane of the leg coordinate system), the mFA and mTA are not necessarily in line with each other. Because of this, the mechanical leg axis (yellow) is not parallel to the mFA or mTA.

Leg coordinate system

23. To define the leg coordinate system, it is a prerequisite that the knee is extended, meaning that the mechanical femoral and tibial axes are parallel to each other (in the sagittal plane of the leg coordinate system).

    AGREE / DO NOT AGREE

    REMARKS:

24. The origin is coincident with the distal femoral joint center (Figure 19).

    AGREE / DO NOT AGREE

    REMARKS:

25. The distal-proximal axis (y-axis) is parallel to the mechanical leg axis (Figure 19).

AGREE / DO NOT AGREE

REMARKS:

26. The medial-lateral axis (z-axis) is parallel to the projection of the medial-lateral axis of the distal femur on the leg axial plane, in the direction of the mechanical leg axis (Figure 19).

AGREE / DO NOT AGREE

REMARKS:

27. The posterior-anterior axis (x-axis) is orthogonal to the y- and z-axis (Figure 19).
AGREE / DO NOT AGREE

REMARKS:

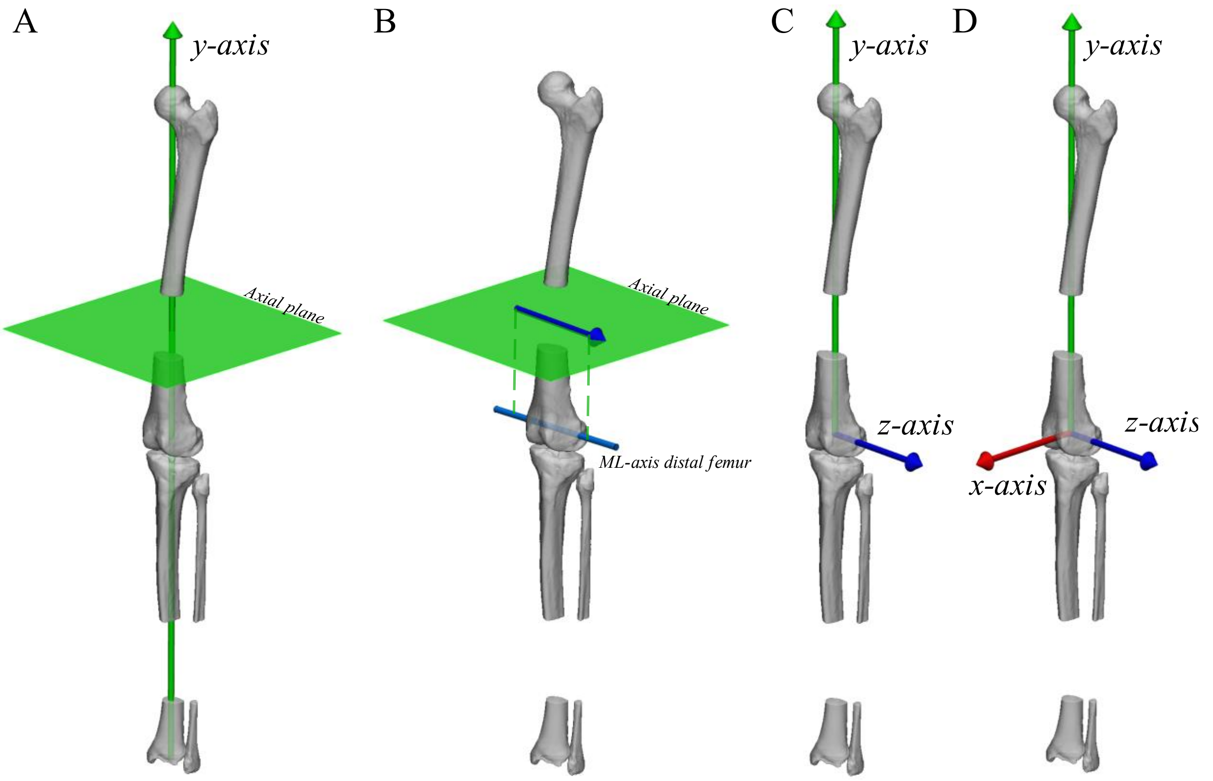

Figure 19: Leg coordinate system. A: the direction of the proximal-distal axis (y-axis; green arrow) is defined parallel to the mechanical leg axis, thereby also defining the axial plane (green plane). B: the direction of the medial-lateral axis (z-axis; blue arrow)

is defined parallel to the medial-lateral axis of the distal femur (cyan cylinder), projected on the axial plane in the direction of the mechanical leg axis (green dashed). C: resulting y- and z-axis defined from the origin (distal femoral joint center). D: the posterior-anterior axis (x-axis; red arrow) follows from being orthogonal to both the y- and z-axis.

Abbreviations: ML, medial-lateral.

QUESTIONNAIRE TO PART 5 OF 5 OF THE SURVEY

Femoral Version and Tibial Torsion

Torsion represents the rotational difference between the distal and the proximal medial-lateral joint orientation around a longitudinal axis [2].

The current gold standard 2D femoral version analysis is based on the central axis through the femoral head and collum as the medial-lateral orientation of the proximal femur, and the femoral posterior condylar axis as the medial-lateral orientation of the distal femur. And 2D tibial torsion analysis is based on the intermalleolar axis as the medial-lateral orientation of the distal tibia/fibula, and the tibial posterior condylar axis as the medial-lateral orientation of the proximal tibia [2].

In contrast, for 3D torsion analysis in the tibia/fibula, the ISB has recommended that the medial-lateral orientation of the proximal joint coincides with the coronal plane of the tibial/fibular coordinate system, which, in turn, is based on a central medial-lateral axis. And the medial-lateral orientation of the distal tibial/fibular joint is recommended to coincide with the intermalleolar axis (alike the 2D alignment analysis) [3]. Although the ISB did not recommend on 3D version analysis in the femur, it can be deduced from the definition in the tibia/fibula. The medial-lateral orientation of the distal femoral joint then coincides with the coronal plane of the femoral coordinate system, which, in turn, is based on a central medial-lateral condylar axis. And the medial-lateral orientation of the proximal femoral joint is then defined by the central axis through the femoral head and collum (alike the 2D analysis). Thus, in 3D malalignment analysis based on a 3D bone model, femoral version and tibial torsion are based on a central and not posterior condylar distal femoral and proximal tibial axis, respectively. And since the femoral and tibial central medial-lateral joint orientation axes and posterior condylar axes do not necessarily align when projected on the axial reference plane, 2D and 3D torsion parameter values might differ.

Furthermore, as was the case for the femoral and tibial/fibular coordinate systems, of contention is the ISB's reliance on single datapoint landmarks only (similar to the 2D alignment analysis method) [3], while in 3D bone models there is access to a multitude of points in the available surface data.

In the fifth and last part, principles on how to derive proximal and distal medial-lateral femoral and tibial joint orientations from their respective 3D bone models are proposed. With this information, distal and proximal medial-lateral joint orientations can be projected on axial anatomical reference planes of their respective coordinate system so that they can be expressed as femoral version or tibial torsion.

Proximal Medial-Lateral Femoral Joint Orientation

28. For femoral version, the medial-lateral joint orientation of the proximal femur is based on a line connecting the proximal femoral joint center to the center of the femoral neck (Figure 20).

    AGREE / DO NOT AGREE

    REMARKS:

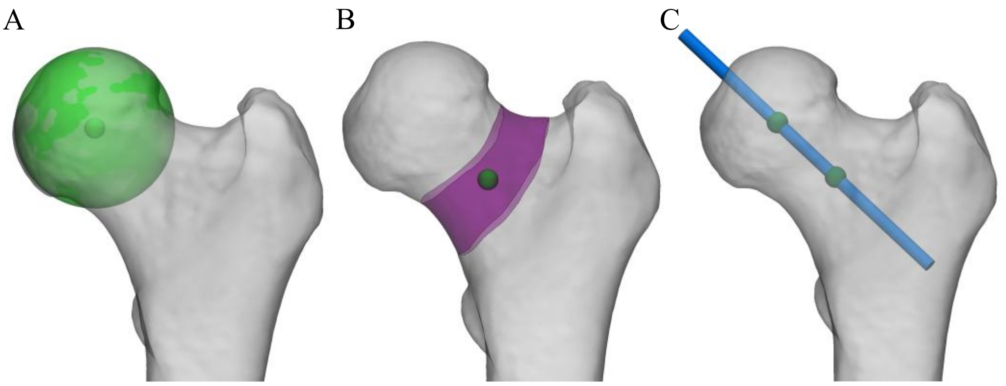

Figure 20: Possible method of how to obtain the medial-lateral axis of the proximal femur, anterior-posterior view. A: femoral head center could be obtained by finding the centroid (green) of a sphere (green) fit to the identified articular surface of the femoral head. B: femoral neck center could be obtained by finding the centroid (dark green) of all available surface data of the femoral neck (purple). C: by connecting the femoral head center (green) and the femoral neck center (dark green) the neck-femur orientation could be obtained (blue).

Distal Medial-Lateral Femoral Joint Orientation

29. For femoral version, the medial-lateral joint orientation of the distal femur is based on the central distal femoral axis which is derived from all available surface data of the articular surface of the medial and lateral distal femoral condyles (Repeat Figure 15).

    AGREE / DO NOT AGREE

    REMARKS:

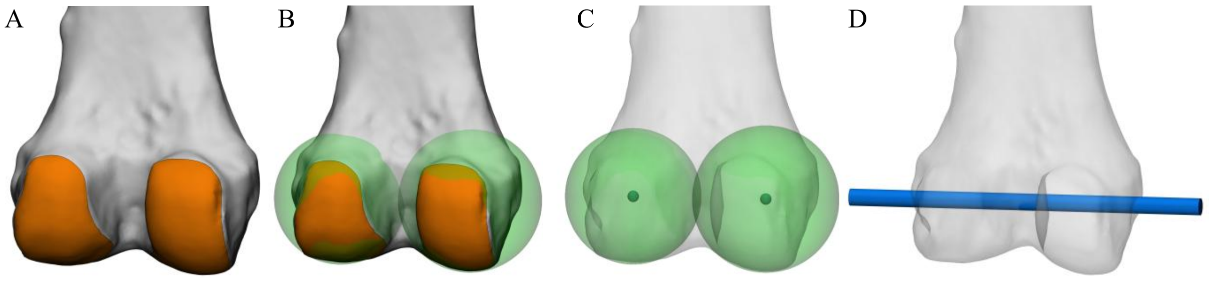

Repeat Figure 15: Possible method of how to obtain the medial-lateral axis of the distal femur, posterior-anterior view. A: articular surfaces of the medial and lateral condyles (orange). B: best fit spheres to each articular surface (green). C: resulting centroids from the fit spheres (dark green). D: the medial-lateral axis of the distal femur could be obtained by connecting the centroids.

Proximal Medial-Lateral Proximal Tibial Joint Orientation

30. For tibial torsion, the medial-lateral joint orientation of the proximal tibia is based on the central proximal tibial axis which is derived from all available surface data of the articular surface of the medial and lateral tibial plateau (Repeat Figure 17).

    AGREE / DO NOT AGREE

    REMARKS:

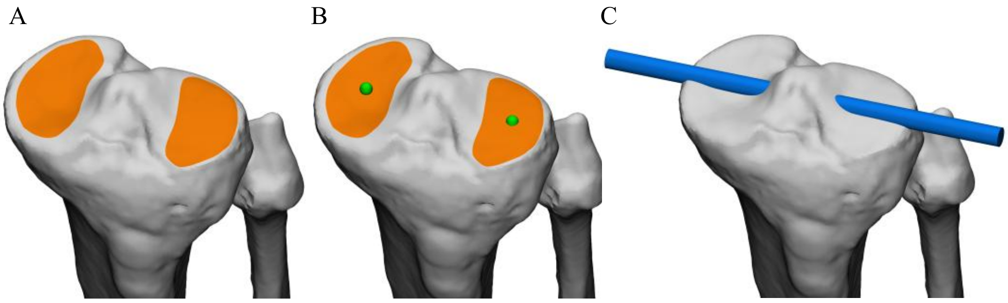

Repeat Figure 17: Possible method of how to obtain the medial-lateral axis of the proximal tibia, anterolateral view. A: articular surfaces of the medial and lateral tibial plateau (orange). B: resulting centroids of each articular surface (green). C: the medial-lateral axis of the proximal tibia (blue) could be obtained by connecting the centroids.

Distal Medial-Lateral Tibial/fibular Joint Orientation

31. For tibial torsion, the medial-lateral joint orientation of the distal tibia/fibula is based on the intermalleolar axis which is derived from all available surface data the articular surfaces of the medial and lateral malleolus (Figure 21).

AGREE / DO NOT AGREE

REMARKS:

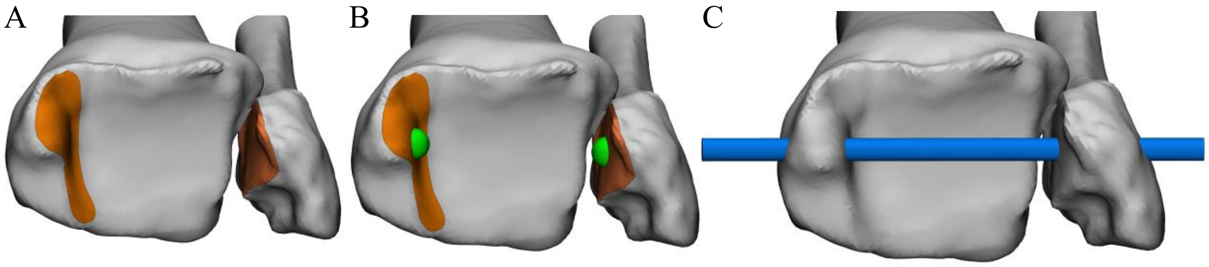

Figure 21: Possible method of how to obtain the medial-lateral distal tibial/fibular axis, distal view. A: identification of the articular surface of the medial and lateral malleolus (orange). B: identification of the centroids of the articular surfaces of the medial and lateral malleolus (green). C: by connecting the centroids, the intermalleolar axis could be obtained.

REFERENCES

1. Grood ES, Suntay WJ (1983) A joint coordinate system for the clinical description of three-dimensional motions: application to the knee. J Biomech Eng. May:136-144
2. Paley D. *Principles of Deformity Correction.* 10.1007/978-3-642-59373-4: Springer Berlin; 2002.
3. Wu G, Siegler S, Allard P, Kirtley C, Leardini A, Rosenbaum D, et al. (2002) ISB recommendation on definitions of joint coordinate system of various joints for the reporting of human joint motion - part I: ankle, hip, and spine. Journal of Biomechanics 35:543-548

A2. Round 4 complete survey

General Introduction

First of all, thank you for your valuable input in the previous round of this Delphi survey.

Thirty-six respondents fully completed the survey, with 26/31 (84%) of statements reaching an agree percentage of > 80%, which we defined as closing criterium beforehand. This leaves five statements to be discussed again, with an added reflection to your provided comments and remarks. After a general response to common returning themes throughout the feedback, you will be asked to respond to the five remaining statements again, each one with an introduction as a response to the provided feedback. Your time investment is expected to be about 10 minutes for this survey.

General response to feedback

One common feedback point was the need for more context to be able to agree with a statement. As correctly pointed out, a principle to alignment analysis might be different when comparing anatomical alignment versus functional alignment, and might be dependent on the data available. Therefore, we would like to clarify the context and expand on the presented rationale in the first round of the survey:

As stated in the rationale previously, the **clinical context** of this survey is in osteotomies around the knee and total knee arthroplasty. Specifically, deformity analysis of a native leg with the goal of **alignment correction** or **TKA component alignment**.

Thus, the context of this survey, is that we present **principles** of **bony** alignment analysis on **3D bone models**, **irrespective of the imaging modality**.

In the future, we might move to functional/dynamic alignment analysis based on functional joint centers and joint orientations, possibly including gait/kinematic analysis, but that lies beyond the scope of this survey. As a first step, consensus on the principles of bony alignment analysis on 3D bone models must be reached.

The five statements not reaching consensus were:
1. Distal Femoral Joint Center
2. X-, Y-, Z-axis nomenclature
3. Knee extension prerequisite for leg coordinate system
4. Femoral version distal femur
5. Tibial torsion proximal tibia

Because of the similarity between the received feedback of statements 4 and 5 (version/torsion), they are combined on one page with one introduction.

Distal Femoral Joint Center

Original statement
"The distal femoral joint center is derived from all available surface data of the articular surface of the medial and lateral distal femoral condyles."

Agree: 26/36 (72%)
Disagree: 10/36 (28%)

Respondents' comments (part 1)
A part of the respondents who disagreed with the stated principle commented on the presented method in the figure, and suggested a different method such as a cylinder fit.

Reflection on the comments (part 1)
These different methods still follow the same principle: use of all available surface data of the articular surface of the medial and lateral distal femoral condyles. This would in fact agree with the asked principle. You can apply different methods based on all available articular surface data. Examples: 1. Two sphere fits 2. Center of mass of both articular surfaces 3. Cylinder fit.

Respondents' comments (part 2)
Another part of the respondents who disagreed with the stated principle suggested to use a single datapoint on the 3D bone model's surface as the distal femoral joint center, alike the current 2D framework.

Reflection on the comments (part 2)
In the 3D literature, 2D principles are still used, discarding a vast amount of information of the 3D bone model's surface data [1]. In this respect, several respondents quoted the anterior point of the top of the notch [2] as the distal femoral joint center. However, next to this option, many other options were also found in a systematic review of the literature. Nevertheless, in 2D, although the distal femoral joint center was defined as the top of the notch in the coronal plane, it was also defined as the point midway the anterior and posterior cortex of the femoral condyles in the sagittal plane [3]. So even in 2D, the distal femoral joint center is defined in the middle of the femoral condylar block (and not as the most anterior point of the top of the femoral notch) (Figure 1). Instead of using a single datapoint, on a 3D bone model's surface, all available articular surface data of the femoral condyles could be used to define the distal femoral joint center in the middle of the femoral condylar block.

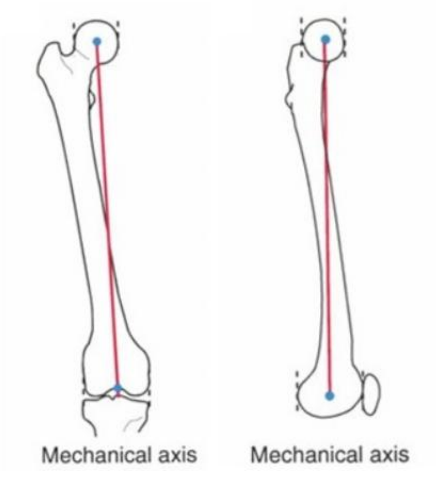

Figure 1: Definition of the distal femoral joint center in 2D by Paley et al. Left: in the coronal plane, the distal femoral joint center is defined at the top of the condylar notch. Right: in the sagittal plane, the distal femoral

joint center is defined as the midpoint between the anterior and posterior condylar cortex. Image adapted from Paley et al. [3]

After these reflections on the provided comments, we ask whether you can agree with the original statement to find consensus:

Q2 The distal femoral joint center is derived from all available surface data of the articular surface of the medial and lateral distal femoral condyles.

Agree

Disagree

Q3 Comments / Remarks regarding Q2 (optional)

References

[1] Veerman, Q. W. T., ten Heggeler, R. M., Tuijthof, G. J. M., Graaff, F., Fluit, R. & Hoogeslag, R. A. G. (2024) High variability exists in 3D leg alignment analysis, but underlying principles that might lead to agreement on a universal framework could be identified: A systematic review. Knee Surgery, Sports Traumatology, Arthroscopy, 1–15. https://doi.org/10.1002/ksa.12512

[2] Victor J, Van Doninck D, Labey L, Innocenti B, Parizel PM, Bellemans J. How precise can bony landmarks be determined on a CT scan of the knee? Knee. 2009 Oct;16(5):358-65. doi: 10.1016/j.knee.2009.01.001

[3] Paley D. Principles of Deformity correction.10.1007/978-3-642-59373-4: Springer-Verlag Berlin Heidelberg New York; 2002.

Nomenclature X-, Y-, and Z-axis

Original statement

"Conform the ISB recommendations, the distal-proximal axis is the y-axis, and points cranially; the medial-lateral axis is the z-axis, and points laterally; and the posterior-anterior axis is the x-axis, and points anteriorly."

Agree: 28/36 (78%)
Disagree: 8/36 (22%)

Respondents' comments

Both agreeing and disagreeing respondents presented valuable background information as to why the originally presented statement might not be correct in the current context that we wish to use them:

· The ISB recommendation was largely based on the context of gait analysis, in which the primary gait direction (forward) was defined as x-axis. This ISB recommendation may thus not be suited for the anatomical coordinate system that we describe here.
· Several respondents pointed out that the presented statement is not congruent with how coordinate systems are defined in for example a CT-scanner or 3D software like 3-matic.
· To ensure a right-hand coordinate system and prevent confusion between left and right legs, the medial-lateral axis should always point to the right (and not laterally as previously stated).

Reflection on the comments

Based on the remarks, we turn to Grood and Suntay's [1] definition that is congruent with current medical software, and have revised the statement based on your input.

Thus, we ask whether you can agree with this revised statement to find consensus:

Q4 The medial-lateral axis is the x-axis, and points to the right; the posterior-anterior axis is the y-axis, and points anteriorly; and the distal-proximal axis is the z-axis, and points cranially.

Agree

Disagree

Q5 Comments / Remarks regarding Q4 (optional)

References

1. Grood ES, Suntay WJ (1983) A joint coordinate system for the clinical description of three-dimensional motions: application to the knee. J Biomech Eng. May:136-144

Knee extension prerequisite for leg coordinate system

Original statement
"To define the leg coordinate system, it is a prerequisite that the knee is extended, meaning that the mechanical femoral and tibial axes are parallel to each other (in the sagittal plane of the leg coordinate system)."

Agree: 28/36 (78%)
Disagree: 8/36 (22%)

Respondents' comments
The respondents who disagreed highlighted that expecting knee extension is not clinically viable, because it does not consider deformities or fixed flexion or extension deficits.

Reflection on the comments
Indeed, the leg CS can, of course, be defined with the knee in any flexion angle. After reading the provided comments, we realized the original statement was inaccurate for what we tried to define. We meant to ask whether true values for distal femoral and proximal tibial joint orientation angles (i.e., the projections of the distal femoral and proximal tibial joint orientation and the relevant longitudinal axes on the coronal plane of the leg coordinate system) can only be calculated with the knee in extension.

In the 2D framework, it was already defined that leg extension is a prerequisite for proper coronal plane leg alignment analysis on weight-bearing leg radiographs [1]. And for the 2D as well as for a 3D framework, it was already reported that flexion and rotation of the knee influences coronal plane alignment parameters values [2,3].

Additionally, as also pointed out by a respondent, 3D allows us to virtually rotate the single bones in the sagittal plane until this prerequisite is obtained, before measuring coronal alignment in a leg coordinate system [3]. Furthermore, although any deformities leading to the inability of the patient to extend the knee can be analyzed much better on 3D bone models compared to 2D radiographs and CT-slices, the abnormal results for distal femoral and proximal tibial joint orientation angles in the coronal plane of the leg of a flexed knee have to be considered.

Thus, we ask whether you can agree with this revised statement to find consensus:

Q6 To define distal femoral and proximal tibial joint orientation angles (i.e., the projection of the distal femoral and proximal tibial joint orientation and the relevant longitudinal axes on the coronal plane of the leg coordinate system), it is a prerequisite that the knee is extended; meaning that the mechanical femoral and tibial axes are parallel to each other (in the sagittal plane of the leg coordinate system).

Agree

Disagree

Q7 Comments / Remarks regarding Q6 (optional)

References

1. Paley D. Principles of Deformity correction.10.1007/978-3-642-59373-4: Springer-Verlag Berlin Heidelberg New York; 2002.
2. Brouwer RW, Jakma TS, Brouwer KH, Verhaar JA. Pitfalls in determining knee alignment: a radiographic cadaver study. J Knee Surg. 2007 Jul;20(3):210-5. doi: 10.1055/s-0030-1248045
3. Brunner J, Jörgens M, Weigert M, Kümpel H, Degen N, Fuermetz J. Significant changes in lower limb

alignment due to flexion and rotation-a systematic 3D simulation of radiographic measurements. Knee Surg Sports Traumatol Arthrosc. 2023 Apr;31(4):1483-1490. doi: 10.1007/s00167-022-07302-x

Femoral version distal femur & Tibial torsion proximal tibia

Original statement (distal femur)
"For femoral version, the medial-lateral joint orientation of the distal femur is based on the central distal femoral axis which is derived from all available surface data of the articular surface of the medial and lateral distal femoral condyles".

Agree: 27/36 (75%)
Disagree: 9/36 (25%)

Original statement (proximal tibia)
"For tibial torsion, the medial-lateral joint orientation of the proximal tibia is based on the central proximal tibial axis which is derived from all available surface data of the articular surfaces of the medial and lateral tibial plateau."

Agree: 28/36 (78%)
Disagree: 8/36 (22%)

Respondents' comments

Some respondents disagreed and suggested to use the current 2D standard of a posterior condylar line on a 3D bone model's surface as the medial-lateral joint orientation of the distal femur for femoral version; and the proximal tibia for tibial torsion.

Reflection on the comments
As stated earlier, femoral version and tibial torsion are defined to represent the rotational difference between the distal and the proximal medial-lateral joint orientation around a longitudinal axis [1].

On 2D CT slices, for the femur, this is defined as the angle between the femoral neck and the coronal plane [1]. And for the tibia/fibula, this is defined as the angle between the intermalleolar axis and the frontal plane of the tibia/fibula. [1]

While for the proximal femur and the distal tibia/fibula the medial-lateral direction are defined by central axes [1], for the distal femur and the proximal tibial medial-lateral direction, the posterior condylar line is often used for the direction of the (medial-lateral) 'coronal plane'. However, the posterior condylar line can be considered a proxy for this, since on the long leg standing radiograph, which in fact defines the leg's coronal plane, the medial-lateral direction of the coronal plane is not defined to be parallel to the posterior condylar line of the femur or the tibia, but to the medial-lateral flexion-extension axis of the knee, which is a central (and not: posterior condylar) axis.

Moreover, in 3D, the ISB (also) defines the medial-lateral joint orientation of the distal femur and proximal tibia to coincide with the coronal plane, which is based on the respective central (and not: posterior condylar) medial-lateral axes (and not on the posterior condylar axis) [2].

The fact that within the 2D framework the posterior condylar axis - instead of the central axis- is used as a proxy is likely because acquiring a posterior condylar axis on 2D CT slices is easier and more robust than acquiring a central axis. However, on 3D bone models, contrary to 2D CT slices, it would be much easier to use all relevant surface data of the distal femur and the proximal tibia to define their medial-lateral direction, which naturally results in central (and not posterior condylar) axes.

Of course, for reasons to improve insight in the relationship between current 2D and (newly to form) 3D

reference values for femoral version and tibial torsion, additional measurements based on a posterior condylar axis can still be used as an additional measurement to the principle proposed here. More in general, the statement(s) in this consensus study are by no means meant to be restrictive to define all sorts of additional alignment parameters, but are merely meant to find consensus on definitions that form the backbone to alignment analysis on 3D bone models.

After this reflection on the provided comments, we ask whether you can agree with the original statements to find consensus:

Q8 For femoral version, the medial-lateral joint orientation of the distal femur is based on the central distal femoral axis which is derived from all available surface data of the articular surface of the medial and lateral distal femoral condyles.

Agree

Disagree

Q9 Comments / Remarks regarding Q8 (optional)

Q10 For tibial torsion, the medial-lateral joint orientation of the proximal tibia is based on the central proximal tibial axis which is derived from all available surface data of the articular surfaces of the medial and lateral tibial plateau.

Agree

Disagree

Q11 Comments / Remarks regarding Q10 (optional)

References

1. Paley D. Principles of Deformity correction. 10.1007/978-3-642-59373-4: Springer-Verlag Berlin Heidelberg New York; 2002.

2. Wu G, Siegler S, Allard P, Kirtley C, Leardini A, Rosenbaum D, et al. (2002) ISB recommendation on definitions of joint coordinate system of various joints for the reporting of human joint motion - part I: ankle, hip, and spine. Journal of Biomechanics 35:543-548
